# Recalibration of deep learning models for abnormality detection in smartphone-captured chest radiograph

**DOI:** 10.1038/s41746-021-00393-9

**Published:** 2021-02-15

**Authors:** Po-Chih Kuo, Cheng Che Tsai, Diego M. López, Alexandros Karargyris, Tom J. Pollard, Alistair E. W. Johnson, Leo Anthony Celi

**Affiliations:** 1 Laboratory for Computational Physiology, Massachusetts Institute of Technology, Cambridge, MA USA; 2 Department of Computer Science, National Tsing Hua University, Hsinchu, Taiwan; 3 Department of Biomedical Informatics, Harvard Medical School, Boston, MA USA; 4 Telematics Department, University of Cauca, Popayán, Cauca Colombia; 5 IBM Research, San Jose, CA USA; 6 Division of Pulmonary Critical Care and Sleep Medicine, Beth Israel Deaconess Medical Center, Boston, MA USA; 7 Department of Biostatistics, Harvard T.H. Chan School of Public Health, Boston, MA USA

**Keywords:** Computer science, Radiography, Biomedical engineering, Image processing, Machine learning

## Abstract

Image-based teleconsultation using smartphones has become increasingly popular. In parallel, deep learning algorithms have been developed to detect radiological findings in chest X-rays (CXRs). However, the feasibility of using smartphones to automate this process has yet to be evaluated. This study developed a recalibration method to build deep learning models to detect radiological findings on CXR photographs. Two publicly available databases (MIMIC-CXR and CheXpert) were used to build the models, and four derivative datasets containing 6453 CXR photographs were collected to evaluate model performance. After recalibration, the model achieved areas under the receiver operating characteristic curve of 0.80 (95% confidence interval: 0.78–0.82), 0.88 (0.86–0.90), 0.81 (0.79–0.84), 0.79 (0.77–0.81), 0.84 (0.80–0.88), and 0.90 (0.88–0.92), respectively, for detecting cardiomegaly, edema, consolidation, atelectasis, pneumothorax, and pleural effusion. The recalibration strategy, respectively, recovered 84.9%, 83.5%, 53.2%, 57.8%, 69.9%, and 83.0% of performance losses of the uncalibrated model. We conclude that the recalibration method can transfer models from digital CXRs to CXR photographs, which is expected to help physicians’ clinical works.

## Introduction

Chest X-ray (CXR) is an essential tool to detect pulmonary abnormalities and has become one of the most prescribed medical tests. An estimated 110 million CXRs are performed annually in the United States^1^, with only around 39,000 radiologists providing the official reading^2^. The need for immediate interpretation or a “wet” read by those who ordered them has prompted clinicians to resort to teleconsultation, especially in settings where they may not have access to a radiologist 24/7. With advances in smartphone technology, doctors have increasingly taken photographs of CXRs and sent them to colleagues for instantaneous reading^3,4^.

In recent years, deep learning algorithms have been proposed as computer-aided diagnosis (CAD) solutions to the radiologist shortage^5–14^. Mostly built on convolutional neural networks (CNNs), the algorithms can detect certain pulmonary abnormalities in CXR images within a second. Numerous studies have shown the competency of CNNs achieving performance close to radiology experts^7,11,12,15–17^.

On the other hand, incorporating the algorithm for automated CXR radiological finding detection into a smartphone offers a number of benefits. First, it will provide access to radiologist-level expertise to a healthcare worker seeking assistance with CXR interpretation or a second opinion anytime, anywhere. Second, it can scale and standardize the process of teleconsultation with less variation in the interpretation compared to one given by different individuals with varying levels of expertise. Third, there is an opportunity for quality assurance as the algorithms can be continuously evaluated and recalibrated against radiologists.

In this study, we explore combining the power of deep learning and the ubiquity of smartphones for CXR finding detection. To the best of our knowledge, this is the first study that recalibrates deep learning models specifically targeting CXR photographs. The target user of the software is a healthcare provider in a resource-limited setting who may not be confident about her/his interpretation or is not a specialist in radiology. It will be easier to install an algorithm on smartphones rather than a legacy computer system in a public hospital or clinic where data interoperability is almost always a challenge. The methodology can also be applied to the abnormality detection on plain films but requires images of the plain films for the recalibration.

We begin the study by showing that the performance of the original CNN-based models trained on high-resolution digital CXR images decreases on CXR photographs. Using less than 200 photographs of CXR, we recalibrated the training process of the models and obtained significant performance improvement. To ascertain the generalizability of the recalibrated model, we measured the performance on four photograph datasets derived from two large and publicly accessible digital CXR databases (MIMIC-CXR^18^ and CheXpert^18,19^). To simulate real-world teleconsultation, these photographs were taken by twelve users including nine medical residents using different computer monitors and smartphones to display and photograph the CXRs. We are also open-sourcing these photograph datasets to the community to promote novel research and the development of similar systems.

## Results

### Experiment design

**Fig. 1:**
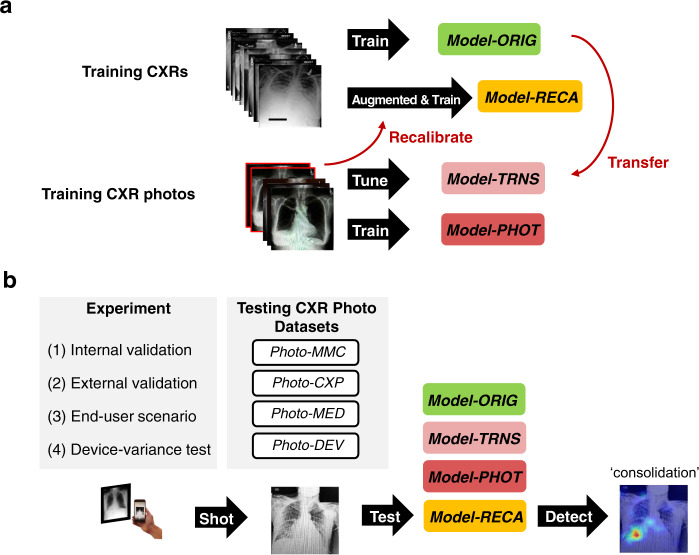
Overview of the proposed method. **a** The uncalibrated model (*Model-ORIG*) was trained on the original CXR images. The transfer learning-based model (*Model-TRNS*) was transferred from the uncalibrated model and fine-tuned by real photographs. The photograph-based model (*Model-PHOT*) was trained by the real smartphone-captured photographs. The recalibrated model (*Model-RECA*) was recalibrated from Model-ORIG by using augmented CXR images. **b** *Model-ORIG*, *Model-TRNS*, *Model-PHOT*, and *Model-RECA* were tested on four CXR photograph datasets (*Photo-MMC*, *Photo-CXP*, *Photo-MED*, and *Photo-DEV*) in four experiments. The performance metrics across 14 labels were calculated. Gradient-weighted Class Activation Mapping was employed for diagnostic focus visualization of models.

We conducted four experiments corresponding to four testing CXR photograph datasets, as shown in Fig. 1b: (1) internal validation using 1,759 photographs taken from MIMIC-CXR dataset (*Photo-MMC*); (2) external validation using 1,337 photographs taken from CheXpert CXR dataset (*Photo-CXP*); (3) end-user scenario using 1,337 photographs taken from CheXpert by nine medical residents (*Photo-MED*); (4) device-variance test using 2020 photographs taken from CheXpert by a single physician with different smartphones and computer monitors (*Photo-DEV*).

Four models based on the MIMIC-CXR dataset were constructed and tested, as shown in Fig. 1a. (1) *Model-ORIG* is the conventional model trained on digital CXRs. (2) *Model-RECA* is our recalibrated model trained on augmented CXRs. (3) *Model-TRNS* is the transfer learning model transferred from *Model-ORIG* and fine-tuned by CXR photographs. (4) *Model-PHOT* is the model directly trained on CXR photographs. The details of model construction and dataset collection are described in the “Methods” section.

### Performance evaluation

**Fig. 2:**
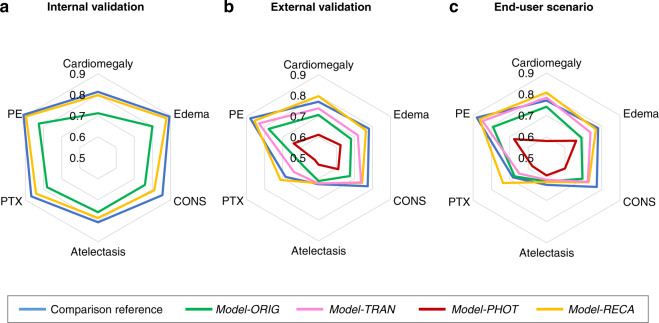
Radiographic detection performance evaluated by AUROCs for six labels including cardiomegaly, edema, consolidation, atelectasis, pneumothorax, and pleural effusion, using different approaches. **a** Internal validation: the comparison for the models tested on MIMIC CXRs and the photographic copies (*Photo-MMC*). **b** External validation: the comparison for the models tested on CheXpert CXRs and the photographic copies (*Photo-CXP*). **c** End-user scenario: the comparison for the models tested on photographs taken by medical residents (*Photo-MED*). In these figures, blue lines show the comparison reference performance of the models tested on original CXRs. Among the three experiments, except for the baseline model, the proposed model, Model-RECA, outperformed the other models. (PTX: Pneumothorax; PE: Pleural effusion; Cons.: Consolidation; *Model-ORIG*: Model trained on MIMIC-CXR; *Model-RECA*: Recalibrated model trained on MIMIC-CXR; *Model-TRNS*: Model transferred from Model-ORIG and fine-tuned by MIMIC-CXR photographs; *Model-PHOT*: Model trained on MIMIC-CXR photographs).

Table 1 and Fig. 2 summarize the results for the first three experiments: internal validation, external validation, and end-user scenario. The areas under the receiver operating characteristic curves (AUROCs) were used to present the performance of different models. Conventional metrics such as sensitivity, specificity, f1 score, and accuracy were also calculated and presented in Supplementary Table 2 - Table 4. Six major radiological findings (cardiomegaly, edema, consolidation, atelectasis, pneumothorax, and pleural effusion) were selected as target labels due to clinical relevance. We obtained the comparison reference by using high-resolution images for both training and testing to avoid the domain discordance problem. The blue lines present the results of comparison reference. The green, yellow, pink, and red lines show the results of the *Model-ORIG*, *Model-RECA*, *Model-TRNS*, and *Model-PHOT*, respectively, using CXR photographs as the testing data.

**Table 1:** AUROCs using MIMIC-based models in internal validation, external validation, and end-user scenario.

	Internal validation			External validation					End-user scenario			
	MIMIC-CXR			CheXpert								
	Comparison reference	*Model-ORIG*	*Model-RECA*	Comparison reference	*Model-ORIG*	*Model-TRNS*	*Model-PHOT*	*Model-RECA*	*Model-ORIG*	*Model-TRNS*	*Model-PHOT*	*Model-RECA*
Cardiomegaly	0.814 ± 0.0108	0.7704 ± 0.0217	0.7986 ± 0.0112	0.8173 ± 0.0193	0.7067 ± 0.0236	0.7385 ± 0.0229	0.6108 ± 0.0247	0.7971 ± 0.0193	0.7414 ± 0.0224	0.7839 ± 0.0206	0.5792 ± 0.0255	0.8081 ± 0.0206
Edema	0.8929 ± 0.012	0.7863 ± 0.0144	0.8775 ± 0.0122	0.7796 ± 0.0144	0.6811 ± 0.0168	0.7187 ± 0.016	0.6226 ± 0.0182	0.7641 ± 0.0155	0.6899 ± 0.0161	0.7397 ± 0.0158	0.6617 ± 0.0172	0.769 ± 0.015
Cons.	0.8549 ± 0.0177	0.7248 ± 0.0299	0.8097 ± 0.0217	0.7564 ± 0.0302	0.675 ± 0.0329	0.7368 ± 0.0292	0.6112 ± 0.0369	0.7425 ± 0.0288	0.6957 ± 0.0355	0.7247 ± 0.0349	0.6024 ± 0.0363	0.7295 ± 0.0317
Atelectasis	0.807 ± 0.0117	0.6383 ± 0.0187	0.7868 ± 0.0123	0.632 ± 0.0194	0.6115 ± 0.0204	0.6235 ± 0.0187	0.5329 ± 0.0205	0.6221 ± 0.0193	0.6108 ± 0.0193	0.6028 ± 0.0193	0.5818 ± 0.0211	0.6136 ± 0.0187
PTX	0.8669 ± 0.0226	0.7587 ± 0.03	0.8408 ± 0.023	0.7266 ± 0.0343	0.5822 ± 0.038	0.6367 ± 0.0408	0.5216 ± 0.0316	0.7119 ± 0.0341	0.6737 ± 0.0376	0.648 ± 0.0399	0.576 ± 0.0361	0.7371 ± 0.0323
PE	0.91 ± 0.0072	0.8686 ± 0.0105	0.8957 ± 0.0077	0.8891 ± 0.0096	0.7781 ± 0.0133	0.8318 ± 0.0121	0.6391 ± 0.0165	0.857 ± 0.0111	0.7914 ± 0.0136	0.8487 ± 0.0115	0.6759 ± 0.0159	0.8645 ± 0.0109

*PTX* pneumothorax, *PE* pleural effusion. *Cons.* consolidation, *Model-ORIG* model trained on MIMIC-CXR, *Model-RECA* recalibrated model trained on MIMIC-CXR, *Model-TRNS* Model transferred from Model-ORIG and fine-tuned by MIMIC-CXR photographs, *Model-PHOT* model trained on MIMIC-CXR photographs.

### Internal validation

**Fig. 3:**
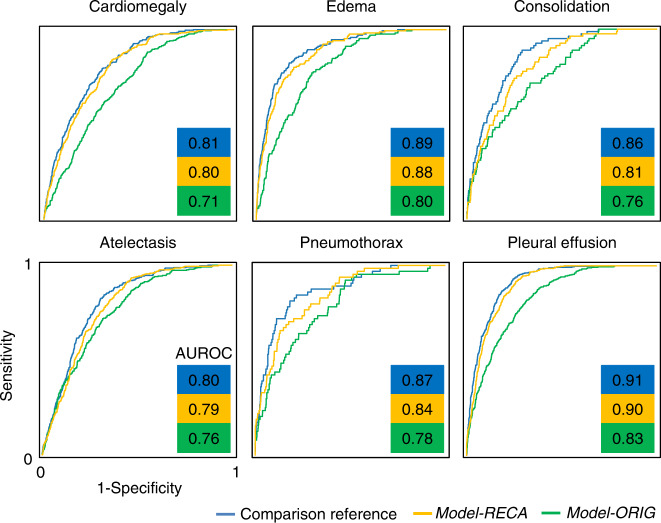
ROC curves for detecting cardiomegaly, edema, consolidation, atelectasis, pneumothorax, and pleural effusion observed in CXRs and CXR photographs. Blue lines show the ROC curves using the uncalibrated model on original CXRs. The yellow and green lines show the results of interpreting CXR photographs using the uncalibrated model (*Model-ORIG*) and recalibrated model (*Model-RECA*), respectively. The AUC of *Model-ORIG* is significantly greater than that of *Model-RECA* for each disease (*p* < 0.0001).

First, we developed and internally tested our models using the MIMIC-CXR database. That is, both the training images and the source of the testing photographs, *Photo-MMC*, were derived from the same database. As shown in Fig. 2a, across the six major radiological findings, *Model-ORIG* shows a performance decrease from an averaged AUROC of 0.86 to 0.77 (*p* < 0.0001) compared to our comparison reference. After model recalibration, the *Model-RECA* shows significant performance recovery from an averaged AUROC of 0.77 to 0.84 (*p* < 0.0001), close to our comparison reference. Figure 3 shows the receiver operating characteristic (ROC) curves. The blue lines show the comparison reference. The yellow lines show the results when the *Model-ORIG* was evaluated using CXR photographs. The green lines show the performance of the *Model-RECA* on the CXR photographs. The AUROC, sensitivity, specificity, F1-score, and accuracy for all 14 labels are presented in Supplementary Table 2. The results reiterate two insights and underscore the importance of this study. First, the model trained on the original CXRs was incapable of maintaining its performance on CXR photographs. Second, the recalibration process improved the model performance and successfully transferred image-based models’ detection accuracy to the CXR photographs.

### External validation

To investigate whether the two insights mentioned above can be generalized to the other database, we tested models developed from the MIMIC-CXR database by the photographs made from an external database, CheXpert. That is, the models were tested on the *Photo-CXP*. As shown in Fig. 2b, across the six radiological findings, the *Model-ORIG* lost its performance from an averaged AUROC of 0.75 to 0.67 when tested on the CXR photographs (*p* < 0.0001). On the other hand, the *Model-RECA* improved an averaged AUROC from 0.67 to 0.75, significantly recaptured the performance loss (*p* < 0.0001). The results of external validation were consistent with those of internal validation. Furthermore, although the transfer learning model (*Model-TRNS*) had significantly better performance than *Model-PHOT* (0.71 vs. 0.59, *p* < 0.0001) and *Model-ORIG* (0.71 vs. 0.67, *p* < 0.0001), the *Model-RECA* still outperformed *Model-TRNS* (0.75 vs. 0.71, *p* < 0.0001). The results imply that although transfer learning strategy can help to deal with the domain shifting problem, the recalibration process provides a better solution for radiographic finding detection on CXR photographs. Finally, when comparing Fig. 2a with Fig. 2b, the difference between the MIMIC-CXR and the CheXpert databases led to AUROC drops for each model and each label, except for the pleural effusion. The AUROC, sensitivity, specificity, F1-score, and accuracy for all 14 labels can be found in Supplementary Table 3.

### End-user scenario

To simulate model performance when implemented in real clinical practice, the *Photo-MED* dataset was used to test models. Nine medical residents were told to take the pictures on their own smartphones and computer monitors as if they would send them to their colleagues for further discussion. Figure 2c shows the comparison results. Again, we reached similar results as those in internal or external validation. The recalibrated model (*Model-RECA*) has the best performance among the four models tested (0.75 vs. 0.72, *p* < 0.0001; 0.75 vs. 0.70, *p* < 0.0001; and 0.75 vs. 0.61, *p* < 0.0001). This achievement is the same as that of the comparison reference (0.75 vs. 0.75). The AUROC, sensitivity, specificity, F1-score, and accuracy for all 14 labels can be found in Supplementary Table 4. The results demonstrate that the model improvement is not user-dependent and the recalibrated model has potential to be deployed to the real clinical works.

### Device-variance test

**Fig. 4:**
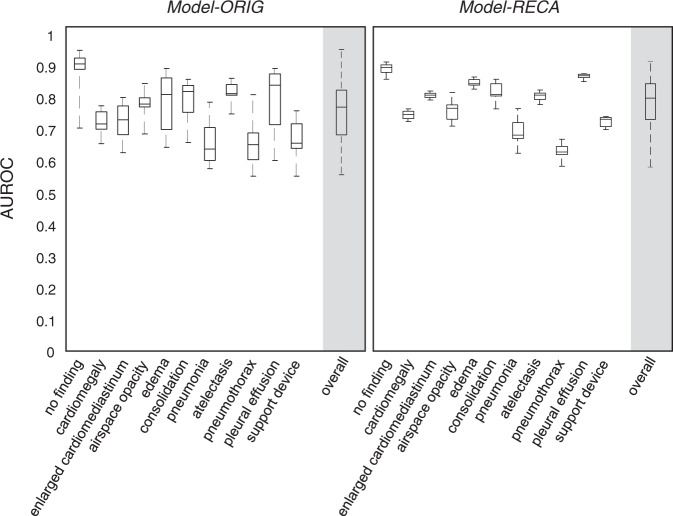
Results of the device-variation experiment, in which the same set of 202 CheXpert CXRs were copied into photographs by ten different device settings. The box plots for the uncalibrated model (*Model-ORIG*) and recalibrated model (*Model-RECA*) show the median and interquartile range of AUROCs. In each box, the central line indicates the median, and the edges of the box indicate the 25th and 75th percentiles. Three labels “fracture,” “lung disease,” and “pleural other” were excluded in the plots because the numbers of cases are less than 1%. The intraclass correlation coefficient (ICC) score for the *Model-ORIG* is [0.39, 0.77] (95% confidence interval) and the ICC for the *Model-RECA* is [0.85, 0.93]. The *p*-value between these two ICCs is smaller than 0.0001, which indicates that *Model-RECA* provides more reliable radiographic detection results.

Figure 4 shows the results of the device-variation test, in which a physician photographed the same set of the CheXpert CXRs ten times by using ten different device setting (smartphones and computer monitors) combinations (*Photo-DEV*). The box plots show the median and interquartile range of AUROCs for *Model-ORIG* and *Model-RECA* across ten settings. The overall AUROC for *Model-RECA* (0.80 ± 0.076) is significantly higher than that for *Model-ORIG* (0.74 ± 0.094) (*p* < 0.0001).

Besides, we used the intraclass correlation coefficient (ICC) to evaluate the radiographic detection stability of both uncalibrated and recalibrated models when tested on photographs taken by different device combinations. The ICC score for the *Model-ORIG*, [0.39, 0.77] (95% confidence interval), is significantly higher than that for the *Model-RECA*, [0.85, 0.93] (*p* < 0.0001). These results indicate that although the noises of photographs generated by different smartphones and computer monitors were various (Supplementary Fig. 1a), the *Model-RECA* can provide more consistent detection results to the same CXR image taken under different noise distribution than *Model-ORIG*.

### Diagnostic visualization

**Fig. 5:**
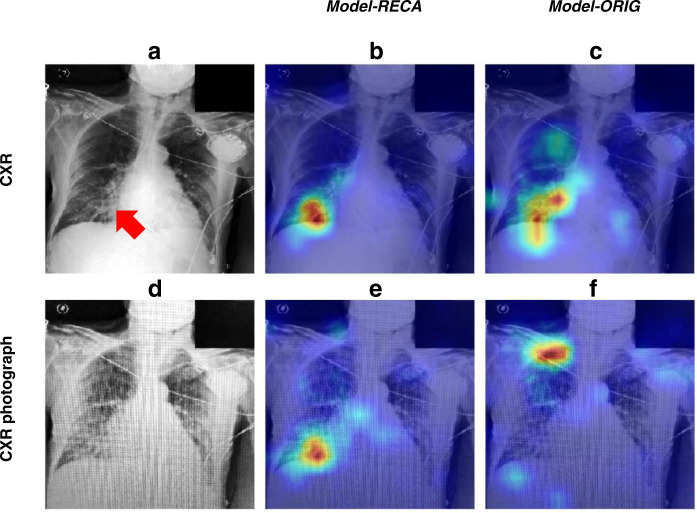
An example of the visualization of the diagnostic focus of two models. **a** An example CXR is diagnosed as consolidation from the radiology report. The red arrow indicates the abnormal location. (**b)** and (**c**) show the diagnostic focus of the recalibrated model (*Model-RECA*) and the uncalibrated model (*Model-ORIG*) tested on the original CXR, respectively. (**e**) and (**f**) show the diagnostic focus of the *Model-RECA* and the *Model-ORIG* tested on the corresponding CXR photograph, respectively. The colors from blue to red map the strengths of the contribution of each image location from low to high for predicting consolidation.

In this study, activation maps were created for demonstration of explainability. By identifying the segment of the CXR that weighed most heavily with regard to the algorithm output, the user is provided some insight of what the algorithm “sees”. This can be particularly useful to determine the trustworthiness of an algorithm’s classification. With the Gradient-weighted Class Activation Mapping (Grad-CAM)^20^, Fig. 5 shows the resilience of *Model-RECA* to noise disturbance in an example case labeled as consolidation. When applied to the original CXR image, irregular opacification at the right lower lobe was correctly tagged by both models. However, when applied to the CXR photograph, *Model-ORIG* was distracted by photography noise and mistakenly used the right clavicle as the determining factor to label consolidation. On the contrary, the *Model-RECA* identified the same location as where it focused when tested on the original CXR images, visually showing its model stability even though the CXR photograph was presented with conspicuous noise. However, the algorithm might be influenced by noise if the quality of the CXR or the photograph captured by the smartphone is suboptimal (see Supplementary Fig. 4).

### Cross-database validation

To re-examine the stability of the recalibration method, we further constructed the CheXpert-based models (*Model-ORIG*, *Model-RECA*, *Model-TRNS*, and *Model-PHOT*) by swapping the roles of MIMIC-CXR and CheXpert databases for training and testing. We again went through all procedures to confirm the consistency of our results and we obtained similar results as shown in Supplementary Fig. 3, Supplementary Table 5, and Supplementary Table. 6.

## Discussion

This study presented a framework to recalibrate conventional deep learning model training process and obtained models capable of detecting radiological findings on CXR photographs. We first demonstrated that the conventional detection algorithms trained on the digital CXRs did not perform well on CXR photographs due to the discordance between images and photographs. We also showed that, from a transfer learning perspective, the performance of the model fine-tuned on a limited number of CXR photographs was not good enough to recover the performance losses. Instead of retraining a model on a large corpus of CXR photographs, we presented a method to recalibrate the models using a small number of photographs from publicly accessible CXR datasets, which saved time from collecting huge number of data for fine-tuning. Finally, we conducted four experiments and showed that the performance losses caused by shifting targets from the original images to photographs could be recovered by the proposed recalibration method.

The main goal of this study is to solve the problem of domain shift in CXR interpretation. Previous research has shown that machine learning systems are vulnerable to adversarial examples generated by smartphone cameras^21^. With different noise, photographs generated from the same image source were classified into incorrect categories. Similarly, our study shows that the uncalibrated model failed to overcome the difference between digital CXRs and CXR photographs. A feasible solution to this obstacle is using transfer learning strategy. A model trained on digital CXRs can be fine-tuned using CXR photographs in order to solve the problem of domain shift. However, we demonstrated that the recalibrated model required only 10% of photographs (*n* = 175) but performed better than the transferred model. Moreover, the proposed recalibration process does not rely on any specific deep learning architecture and thus is applicable to various models. We suggest that the recalibration method can serve as an alternative to transfer learning for the model building when dealing with domain shift problems.

A challenge for recent deep learning advances in radiology is generalizability^22^. Some algorithms with high accuracy, as reported in publications, struggle to translate their success in the real world. A study also demonstrated the reduction in model performance when training and testing were done on different CXR databases, MIMIC-CXR and CheXpert^23^. To ascertain generalizability of our methods, we employed a second large CXR dataset to conduct external validation and experiments with different users and devices. Across these experiments, the performance of the recalibrated model was stably better than the uncalibrated model and close to the comparison reference. Besides, the significantly better ICC of the recalibrated model demonstrated its robustness despite various noise distributions. Finally, we performed a cross-database validation by swapping the training and testing sets of MIMIC-CXR and CheXpert and showed that results were consistent with the analog experiment. These experiments suggest that our recalibrated model has generalizability to different hospitals, users, and devices and can provide a foundation to build a smartphone application to assist clinicians with CXR interpretation anytime, anywhere.

Although a previous work^24^ showed the possibility of directly shifting the models from digital CXRs to CXR photographs, the photographs used to test the model were limited to a small number and certain categories. In our experiments, similar and minor performance loss can only be observed when we internally validated the uncalibrated model with five specific categories (i.e., cardiomegaly, edema, consolidation, atelectasis, and pleural effusion). Otherwise, the uncalibrated model lost its accuracy when tested on an external dataset or more categories of radiological findings.

Phillips and colleagues have also built a CXR photograph dataset^25^, which contains CXR photographs taken by a single physician but using different techniques. Although both studies look at photographs of CXRs, our study focuses on recalibrating the algorithms on images taken by different users and different cameras. The noise generation and non-trivial image transformations of the photographs are greatly affected by camera hardware (e.g., the sensor’s resolution and the construction of the lens) and software (e.g., auto-adjustment of ISO and white balances). Moreover, the photographs taken by a single experienced user could greatly differ from those taken by a less experienced user. Therefore, we built our validation sets by capturing the images using several devices and taken by several users. As shown by our results, the uncalibrated model had greater loss of its accuracy while the recalibrated model performed well when tested on different data sources, users, and devices.

The primary use case envisioned for the smartphone-based algorithm is for assistance with interpretation of a CXR (digital image or plain film) in an acute care facility with a legacy clinical information system. Currently, a messaging application such as WhatsApp is typically employed to take a photo of either the digital image or the plain film and send it to a colleague for a wet read. Is the CXR suggestive of pneumonia? Is there pulmonary edema? The use of the smartphone-based algorithm is not intended for the detection of lung nodules for cancer screening nor for quality assurance of radiologists given that these two tasks require high-resolution images. Despite applications limited to acute care, the software can still help address radiologist shortage in low-resource countries. For example, there are only three radiologists in Botswana for two million people^26^. However, in countries like Botswana, CXR films are still printed instead of digitalized. When radiology consultation is required in remote areas, clinicians send printed films to the capital and receive the reading days, if not weeks later. Applying this smartphone-based application can help to shorten the turnaround time and provide immediate assistance to local clinicians.

Lastly, the model performance should be carefully assessed in clinical scenarios. We used AUROCs to evaluate the discrimination of the models. However, clinicians may care more about precision, or positive predictive value, and recall, or sensitivity. The consequence of missing some CXR finding (false negative) must be balanced with the harm of overcalling it (false positive). For example, if clinicians would like to use the algorithm to screen for pneumonia, then the model with the best recall is preferred over one with the best discrimination. However, if the intent is to help filter referrals from rural health centers and decongest strained tertiary care facilities in the capital, then precision is prioritised over recall.

There are a number of limitations to this work. First, both digital CXR databases used in this study were obtained from patients in the US. Ideally, the model should be recalibrated using photographs obtained from the local population. The model will be used, particularly as the common radiographic findings in such a population will likely differ from those in a US population. Second, the model performance we report is tied to the accuracy and consistency of the CXR labels on MIMIC-CXR and CheXpert. For example, the discrimination between “consolidation,” “pneumonia,” and “opacity” may not be the same across different datasets and will interfere with the recalibration process. This issue can be addressed by harmonizing labels and annotations across the CXR datasets before the recalibration. Finally, we did not compare the re-calibration of the algorithm with reader re-calibration when interpreting the high-resolution DICOM image and the low-resolution photo of the same CXR on a smartphone. In all the experiments, the interpretation of the high-resolution DICOM image was used as the gold standard. Neither a human or an algorithm can compensate for a significant loss of information with a reduction in the image resolution.

In summary, we presented a method to recalibrate deep learning models built on high-resolution digital images to detect radiological findings on smartphone-captured CXR photographs. The recalibrated model achieves similar performance as the original model, and its performance is not significantly affected by variation in devices and operators.

## Methods

### Overview

Figure 1 illustrates the proposed method. We first collected CXRs from two databases and created the CXR photograph datasets by taking smartphone photographs of digital CXR. Instead of taking a large number of photographs, we built a series of augmentation functions to augment the training datasets to be photographic-like CXRs. Hyperparameters of augmentation functions were tuned by comparing the similarity between the augmented results and 175 real photographs. The final augmented CXR photographs were used to train the recalibrated model (*Model-RECA*), as shown in Fig. 1a. Three other models (*Model-ORIG*, *Model-TRNS*, and *Model-PHOT*) were constructed for comparison. Finally, as shown in Fig. 1b, the models were tested on four derivative CXR photograph datasets (*Photo-MMC*, *Photo-CXP*, *Photo-MED*, *and Photo-DEV*) corresponding to four experiments (internal validation, external validation, end-user scenario, and device-variance test. The performance metrics and activation maps for 14 labels representing radiological findings were used to evaluate model performance.

### Data collection and curation

We used frontal-view CXR images from MIMIC-CXR and CheXpert databases^18,19^. MIMIC-CXR contains data from 64,588 patients from the Beth Israel Deaconess Medical Center Emergency Department collected between 2011 and 2016. MIMIC-CXR database v2.0.0 has been de-identified. The institutional review boards of Massachusetts Institute of Technology (No. 0403000206) and Beth Israel Deaconess Medical Center (2001-P-001699/14) both approved the creation of the database for research. Requirement for informed consent was waived because the study did not impact clinical care and all protected health information was removed. A total of 14 labels of radiological findings, as listed in Table 2, were extracted from the radiology reports using the CheXpert and NegBio algorithms^18,27^. Twenty-two images, simultaneously labeled as ‘no finding’ and positive for one of the 14 labels, were excluded in the following analyses. A total of 250,022 frontal-view CXR images were randomly separated into training (*n* = 248,263), and testing (*n* = 1759) sets.

**Table 2:** Numbers of cases for 14 labels in MIMIC-CXR and CheXpert datasets.

	MIMIC-CXR				CheXpert			
	Training		Testing		Training		Testing	
Total	248,263		1759		189,892		1337	
No finding	82,662	33.3%	649	36.9%	16,800	8.85%	200	14.96%
Enlarged cardiomediastinum	7866	3.2%	49	2.8%	9132	4.81%	160	11.97%
Cardiomegaly	48,893	19.7%	356	20.2%	23,273	12.26%	178	13.31%
Airspace opacity	55,648	22.4%	369	21.0%	93,744	49.37%	584	43.68%
Lung lesion	7003	2.8%	55	3.1%	6996	3.68%	45	3.37%
Edema	29,389	11.8%	171	9.7%	49,408	26.02%	309	23.11%
Consolidation	11,732	4.7%	81	4.6%	12,933	6.81%	82	6.13%
Pneumonia	18,325	7.4%	109	6.2%	4653	2.45%	30	2.24%
Atelectasis	49,627	20.0%	333	18.9%	29,526	15.55%	269	20.12%
Pneumothorax	11,610	4.7%	64	3.6%	17,633	9.29%	67	5.01%
Pleural effusion	58,727	23.7%	377	21.4%	76,580	40.33%	383	28.65%
Pleural (other)	2135	0.9%	31	1.8%	2497	1.31%	9	0.67%
Fracture	5016	2.0%	28	1.6%	7391	3.89%	45	3.37%
Support devices	74,247	29.9%	451	25.6%	106,628	56.15%	641	47.94%

CheXpert is a publicly available database collected from Stanford Hospital. The database includes 224,316 CXRs from 65,240 patients. Each CXR was labeled with the presence or absence of 14 pulmonary radiological findings. A total of 191,229 frontal-view CXR images were used and were randomly separated into training (*n* = 189,892), and testing (*n* = 1337) sets. The ratio of the size of the training and testing data is the same (1000:7) for both datasets. Another 202 frontal-view CXR images annotated by three board-certified radiologists and originally designed as a validation set, were included to examine the device variation.

Table 2 summarizes the distribution of the radiological findings in the training and test sets of MIMIC-CXR and CheXpert. Prior to the analysis, all images were normalized by histogram equalization.

To create the CXR photograph datasets, we selected the current generation of smartphones with different camera specifications (see Supplementary Table 1). All photographs were taken under random angles, ambiance factors, and noise disturbance. The alignment of each photograph was automatically adjusted with the Microsoft Office Lens App (Microsoft Corp.) to simulate instructions to users for obtaining the best possible image. We reduced the resolution of the photographs to 320 × 320 pixels after they were captured. Four CXR photograph datasets were created.

1. *Photo-MMC*: Photographs of the CXRs in MIMIC-CXR were captured by three participants using eight different smartphones. The images were displayed on eight different computer monitors. The CXR photographs were taken at different times, locations, and using various lighting sources. A total of 1759 photographs were included in the MIMIC-CXR testing set.
2. *Photo-CXP*: Using the same settings as those to create *Photo-MMC*, a total of 1337 photographs were taken from the CheXpert testing set.
3. *Photo-MED*: 1337 photographs in the CheXpert testing set were separated into nine subsets. Nine medical residents were recruited to take photos of each subset by using their own smartphones and monitors. They were instructed to “take photos as if you want to send them to a radiologist for interpretation.” No other instruction or quality requirement was given.
4. *Photo-DEV*: To examine the effect of the make of the computer monitor and the smartphone, 202 photographs of the CheXpert validation dataset were repeatedly taken by a single physician ten times. For the first nine subsets, nine different device settings were used under the same lighting condition and location. The last subset was taken with a brighter lighting condition. This dataset consists of 2020 photographs in total. Supplementary Fig. 1a shows examples of CXR photographs taken by different device settings.

### Data augmentation

We augmented the training datasets by generating simulated CXR photographs with the hyperparameters determined by photographs for recalibration. Eight common types of noise were embedded in the functions: (1) Gaussian noise, (2) saturation change, (3) overexposure, (4) contrast change, (5) motion blur, (6) moiré pattern, (7) Poisson noise and (8) noise-induced by image compression^28^ (see Supplementary Fig. 2). We used the imgaug 0.4.0 library for Python 3.7.0 to generate noise (1)–(5), (7), and (8)^29^. The moiré pattern was simulated using the Radon and inverse Radon transform^30,31^ from the scikit image library v0.17.dev0^32^. These noise simulation functions were aligned in their occurrence order on the optical path, starting from the computer monitor. An example photograph produced by augmentation functions is shown in Supplementary Fig. 1b. The augmented photograph shows the effects of noise patterns, overexposure, and contrast enhancement in the CXR photograph.

### Hyperparameter optimization

Ten hyperparameters were optimized in the augmentation function with the range: (1) the mean (range: 5–20) and (2) the variance (range: 4–12) of Gaussian noise, (3) the possibility of saturation change (range: 0.5–0.8), (4) the white/yellowish ratio of saturation changes (range: 0.6–0.8), (5) the intensity mean (range: 1–1.4) and (6) the intensity variance (range: 0.2–0.4) of overexposure, (7) the intensity of contrast correction (range: 1.6–2.2), (8) the probability of motion blur (range: 0.2–0.5) (9) the probability of moiré pattern (range: 0.3–0.9) and (10) the lambda of Poisson noise (range: 2–10). Motion intensity was fixed to 5, and the compression rate was set to 30–70%.

A similarity comparison between the CXR photographs and the augmented photographs was performed to determine the value of each hyperparameter in the augmentation functions. The similarity was calculated by using the complex wavelet structural similarity method^33^ and the Bhattacharyya distance of image histogram. We performed hyperparameter optimization using a grid search of reasonable values. 10% of the photographs from *Photo-MMC* were partitioned for tuning the hyperparameters and were excluded from the performance evaluation.

Three different parameter selection approaches were adopted to determine the value of each hyperparameter and evaluate their effectiveness based on the performance of the models. The methods are: (1) randomly selecting hyperparameters from the chosen range. (2) Selected by an author based on his subjective perception of each hyperparameter and (3) similarity comparison. The comparison results are shown in Supplementary Table 7.

### Model construction

Deep learning models were built for detecting radiological findings. The training and testing were performed on the Multiple-GPU Google platform. Tensorflow 2.0 and Keras 2.3 were used for model training. A 121-layer Densely Connected Convolutional Network (DenseNet-121)^34^ with max-pooling was used as the comparison reference model architecture, which was also used in the previous studies^7,8,11,18,35^. The consistent results of the comparison reference model can also be found in recent studies using the same model structure (DenseNet-121) and databases (MIMIC-CXR and CheXpert)^23,35^. The input image size was 320 by 320 because the previous study has demonstrated that performance did not increase with higher resolution CXR images and the use of higher resolution images requires more computational cost^36^. The initial weights of the network were randomly initialized. The final fully connected layer contained 14 outputs corresponding to the 14 target labels. Binary cross entropy was chosen as the loss function and the Adam optimizers were applied in the training process with parameters: learning rate = 0.001, beta1 = 0.9, and beta2 = 0.999^37^. As shown in Fig. 1a, four models were constructed: The comparison reference model, *Model-ORIG*, was trained using the original MIMIC-CXR images. The recalibrated model, *Model-RECA*, was trained using the augmented CXR photographs. The model *Model-TRNS* was acquired by using the *Photo-MMC* dataset (*n* = 1,759) to fine-tune the *Model-ORIG*. Finally, the photograph-based model, *Model-PHOT*, was directly trained on the real photographs in *Photo-MMC* (*n* = 1759).

We trained the *Model-ORIG* and *Model-RECA* using mini-batches of size 32 and five epochs. The models converged after five epochs. We trained the *Model-TRNS* and *Model-PHOT* using 10 and 50 epochs, respectively, and after that the model was converged. For the *Model-ORIG*, the training dataset was augmented by a random transformation (rotating ±7 degrees, scaling ±2%, and shearing ±5 pixels) twice^38^. For the *Model-RECA*, we augmented the training dataset using our augmentation functions with two sets of hyperparameters, which were determined by complex wavelet structural similarity method^33^ and the Bhattacharyya distance of image histogram, respectively. The total numbers of training data were the same for *Model-ORIG* and *Model-RECA* (*n* = 496,570).

### Experiment design

Figure 1b shows that the four models (*Model-ORIG*, *Model-TRNS*, *Model-PHOT*, *and Model-RECA*) described above were tested on four CXR photograph datasets (*Photo-MMC*, *Photo-CXP*, *Photo-MED*, and *Photo-DEV*) separately, which were constructed for the purpose below:

1. Internal validation
  The *Model-ORIG* and *Model-RECA* were tested on the original MIMIC-CXR testing set and *Photo-MMC*. The *Model-TRNS* and *Model-PHOT* were excluded in this experiment because they were trained using *Photo-MMC*.
2. External validation
  CheXpert testing dataset and *Photo-CXP* were used as external datasets to test the performance of four models.
3. End-user scenario
  Four models were tested on the *Photo-MED* to investigate the model performance when applied to real-world healthcare scenarios.
4. Device-variance test

To investigate whether the model performance is device-dependent, *Photo-DEV* was used to test the models.

### Performance evaluation and statistical analysis

We calculated one-versus-all AUROC, sensitivity, specificity, F1-score, and binary classification accuracy in each experiment to evaluate model performance. In the device-variance test, the intraclass correlation coefficient (ICC) was used to evaluate the intra-rater reliability (i.e., the stability of label production in our test) of both models. We used a “two-way mix effect,” “single measurement,” and “absolute agreement” model in R to estimate the final value^32^. Bootstrapping was used to estimate the 95% confidence interval and perform t statistics. Finally, we used a nonparametric approach to estimate the *p*-value. We bootstrapped the testing data 1000 times to obtain the AUROCs and performed the Welch’s two sample *t*-test to calculate the *p*-value.

### Model visualization

Finally, we employed the Grad-CAM^20^ to obtain visual explanations for each label of our CNN-based models. The heatmaps produced by Grad-CAM can be used to visualize the diagnostic focus of the working algorithm and investigate whether the algorithms used the same visual pattern to detect radiological findings as what radiologists have used.

### Cross-database validation

We swapped the roles of MIMIC-CXR and CheXpert datasets for training and testing and then went through all procedures again. The parameters used in the CheXpert-based model construction were the same as those in MIMIC-based model construction. As a result, three additional models were constructed. The baseline model, *Model-ORIG*, was trained by the original CheXpert CXR images. The recalibrated model, *Model-RECA*, was trained by the augmented CXR photographs. The model *Model-TRNS* was acquired by using the *Photo-CXP* dataset (*n* = 1337) to fine-tune the *Model-ORIG*. The photograph-based model, *Model-PHOT*, was trained on the real photographs in *Photo-CXP* (*n* = 1337). These four models were tested on two CXR photograph datasets (Photo-CXP and Photo-MMC), and one-versus-all AUROC, sensitivity, specificity, F1-score, and binary classification accuracy were computed for each label.

### Reporting summary

Further information on experimental design is available in the Nature Research Reporting Summary linked to this paper.

## Supplementary information

Supplementary Information

Reporting Summary

## Data Availability

The CXR photographs used in this study are publicly available on PhysioNet (https://physionet.org/content/cxr-phone/1.0.0/).

## Code availability

The codes are available at https://github.com/PoChihKuo/RECA-CXR.

## References

[1] Mettler FA (2020). Patient exposure from radiologic and nuclear medicine procedures in the United States: procedure volume and effective dose for the period 2006-2016. Radiology.

[2] Rosenkrantz AB, Hughes DR, Richard Duszak J (2016). The U.S. radiologist workforce: an analysis of temporal and geographic variation by using large national datasets. Radiology.

[3] Boissin C, Blom L, Wallis L, Laflamme L (2017). Image-based teleconsultation using smartphones or tablets: qualitative assessment of medical experts. Emerg. Med. J..

[4] Giansanti D (2020). WhatsApp in mHealth: an overview on the potentialities and the opportunities in medical imaging. Mhealth.

[5] Auffermann WF, Gozansky EK, Tridandapani S (2019). Artificial intelligence in cardiothoracic radiology. Am. J. Roentgenol..

[6] McBee MP (2018). Deep learning in radiology. Academic Radiol..

[7] Rajpurkar, P. et al. Chexnet: radiologist-level pneumonia detection on chest x-rays with deep learning. Preprint at https://arxiv.org/abs/1711.05225 (2017).

[8] Baltruschat IM, Nickisch H, Grass M, Knopp T, Saalbach A (2019). Comparison of deep learning approaches for multi-label chest X-ray classification. Sci. Rep..

[9] Taylor, A. G., Mielke, C. & Mongan, J. Automated detection of moderate and large pneumothorax on frontal chest X-rays using deep convolutional neural networks: a retrospective study. *PLoS Medicine* 15, 10.1371/journal.pmed.1002697 (2018). 10.1371/journal.pmed.1002697 PMC6245672 30457991

[10] Annarumma M (2019). Automated triaging of adult chest radiographs with deep artificial neural networks. Radiology.

[11] Rajpurkar P (2018). Deep learning for chest radiograph diagnosis: a retrospective comparison of the CheXNeXt algorithm to practicing radiologists. PLoS Med..

[12] Wang X (2017). ChestX-ray8: hospital-scale chest X-ray database and benchmarks on weakly-supervised classification and localization of common thorax diseases. Proc. IEEE Conf. Computer Vis. Pattern Recognit..

[13] Majkowska A (2020). Chest radiograph interpretation with deep learning models: Assessment with radiologist-adjudicated reference standards and population-adjusted evaluation. Radiology.

[14] Nam JG (2019). Development and validation of deep learning–based automatic detection algorithm for malignant pulmonary nodules on chest radiographs. Radiology.

[15] Lakhani P, Sundaram B (2017). Deep learning at chest radiography: automated classification of pulmonary tuberculosis by using convolutional neural networks. Radiology.

[16] Tang Y-X (2020). Automated abnormality classification of chest radiographs using deep convolutional neural networks. npj Digital Med..

[17] Gündel S (2019). Learning to recognize abnormalities in chest X-rays with location-aware dense networks. Prog. Pattern Recognit. Image Anal. Computer Vis. Appl..

[18] Irvin J (2019). CheXpert: a large chest radiograph dataset with uncertainty labels and expert comparison. Proc. AAAI Conf. Artif. Intell..

[19] Johnson AEW (2019). MIMIC-CXR, a de-identified publicly available database of chest radiographs with free-text reports. Sci. Data.

[20] Selvaraju RR (2017). Grad-CAM: visual explanations from deep networks via gradient-based localization. Proc. IEEE Int. Conf. Computer Vis..

[21] Kurakin, A., Goodfellow, I. & Bengio, S. Adversarial examples in the physical world. *Proc. Workshop Int. Conf. Learn. Represent.* **2017**, 1–11 (2016).

[22] Zech, J. R. et al. Variable generalization performance of a deep learning model to detect pneumonia in chest radiographs: a cross-sectional study. *PLoS Medicine* 15, 10.1371/journal.pmed.1002683 (2018). 10.1371/journal.pmed.1002683 PMC6219764 30399157

[23] Pooch, E. H., Ballester, P. L. & Barros, R. C. Can we trust deep learning models diagnosis? The impact of domain shift in chest radiograph classification. In *Proc. of Machine Learning Research* **121**, 136–155 (2020).

[24] Rajpurkar, P. et al. CheXpedition: Investigating generalization challenges for translation of chest X-ray algorithms to the clinical setting. Preprint at https://arxiv.org/abs/2002.11379 (2020).

[25] Phillips, N. A. et al. CheXphoto: 10,000+ smartphone photos and synthetic photographic transformations of chest X-rays for benchmarking deep learning robustness. In *Proc. of Machine Learning Research* **136**, 318–327 (2020).

[26] Rosman DA, Bamporiki J, Stein-Wexler R, Harris RD (2019). Developing diagnostic radiology training in low resource countries. Curr. Radiol. Rep..

[27] Peng Y (2018). Negbio: a high-performance tool for negation and uncertainty detection in radiology reports. AMIA Summits Transl. Sci. Proc..

[28] Boncelet, C. In *The Essential Guide to Image Processing* (ed Al Bovik) 143–167 (Academic Press, 2009).

[29] Jung, A. imgaug (2017) https://github.com/aleju/imgaug (2019).

[30] Deans, S. R. *The Radon Transform and Some of its Applications* (Courier Corporation, 2007).

[31] Saveljev V, Kim S-K (2012). Simulation and measurement of moiré patterns at finite distance. Opt. Express.

[32] Van der Walt S (2014). scikit-image: image processing in Python. PeerJ.

[33] Sampat MP, Wang Z, Gupta S, Bovik AC, Markey MK (2009). Complex wavelet structural similarity: a new image similarity index. IEEE Trans. image Process..

[34] Huang G, Liu Z, Van Der Maaten L, Weinberger KQ (2017). Densely connectedconvolutional networks In. Proc. IEEE Conf. Computer Vis. Pattern Recognit.

[35] Seyyed-Kalantari, L., Liu, G., McDermott, M. & Ghassemi, M. CheXclusion: Fairness gaps in deep chest X-ray classifiers. Preprint at https://arxiv.org/abs/2003.00827 (2020). 33691020

[36] Sabottke CF, Spieler BM (2020). The effect of image resolution on deep learning in radiography. Radiology: Artif. Intell..

[37] Kingma, D. P. & Ba, J. Adam: A method for stochastic optimization. Preprint at https://arxiv.org/abs/1412.6980 (2014).

[38] Pham, H. H., Le, T. T., Tran, D. Q., Ngo, D. T. & Nguyen, H. Q. Interpreting chest X-rays via CNNs that exploit disease dependencies and uncertainty labels. Preprint at https://arxiv.org/abs/1911.06475 (2019).

