# Supplementary material for: Recalibration of deep learning models for abnormality detection in smartphone-captured chest radiograph
Source: NPJ Digit Med. 2021 Feb 15;4:25. doi: 10.1038/s41746-021-00393-9 (PMC7884693; doi:10.1038/s41746-021-00393-9)
Supplement: Supplementary file 1 — Supplementary Information [file 41746_2021_393_MOESM1_ESM.pdf]

Recalibration of Deep Learning Models for Abnormality Detection in Smartphone-Captured Chest Radiograph

Supplementary Figures

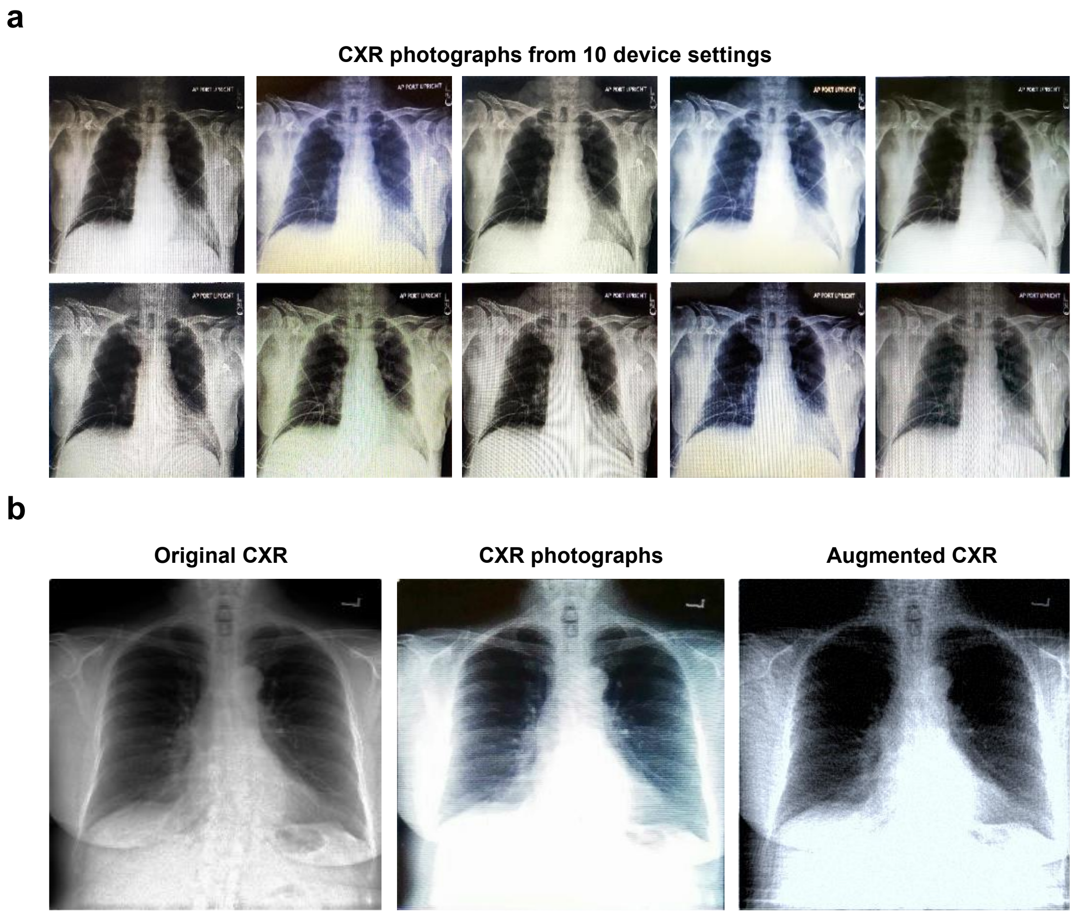

Supplementary Figure 1. Examples of CXR photographs. **a** CXR photographs taken from a same CXR example by 10 different device settings. **b** Examples of the original CXR, CXR photographs, and the augmented CXR photographs. In the photographs, the overexposure blinded detailed structure of the heart and infraphrenic regions. Bilateral upper lung fields were dark due to built-in contrast enhancement of the smartphone camera. The augmented CXR photographs caught these changes.

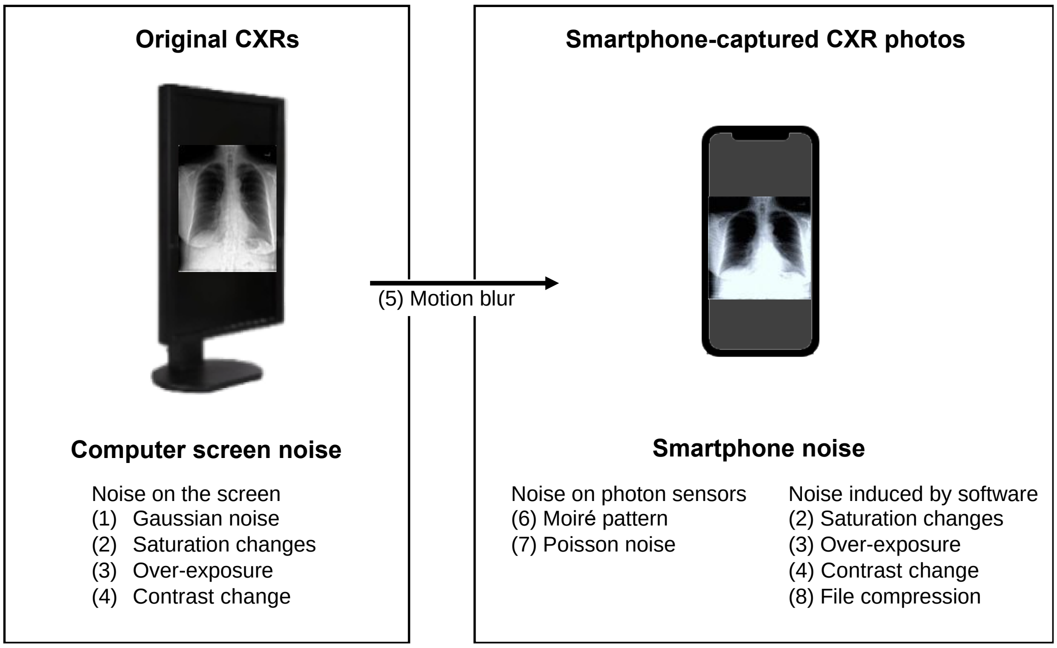

Supplementary Figure 2. The origin of noises simulated in this study. Noise on the screen includes Gaussian noise, saturation change, overexposure, and contrast change. The motion blur occurs during the capture. Smartphone noises can be induced on photons (moiré pattern and Poisson noise) or by software (saturation change, overexposure, contrast change, and file compression).

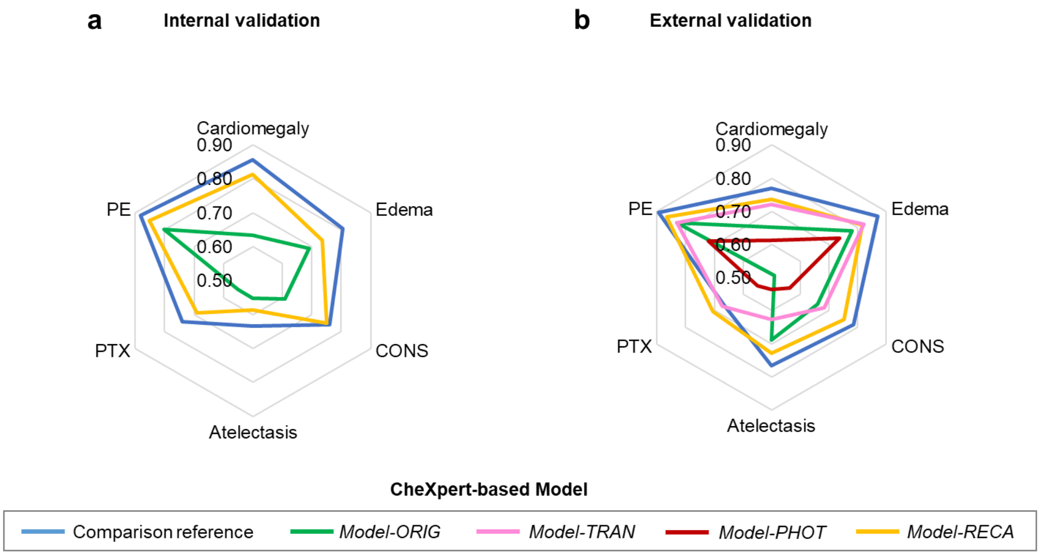

Supplementary Figure 3. Prediction performance of CheXpert-based models evaluated by AUROCs for six labels including cardiomegaly, edema, consolidation, atelectasis, pneumothorax, and pleural effusion, using different approaches. **a** Internal validation: the comparison for *Model-ORIG* tested on CheXpert CXRs, *Model-ORIG* tested on *Photo-CXP*, and *Model-RECA* tested on *Photo-CXP*. **b** External validations: the comparison for *Model-ORIG* tested on MIMIC CXRs, *Model-ORIG* tested on *Photo-MMC*, *Model-TRNS* tested on *Photo-MMC*, *Model-PHOT* tested on *Photo-MMC*, and *Model-RECA* tested on *Photo-MMC*. (PTX: Pneumothorax; PE: Pleural effusion; Cons.: Consolidation; *Model-ORIG*: Model trained on CheXpert CXR; *Model-TRNS*: Model transferred from the *Model-ORIG* and fine-tuned on *Photo-CXP*; *Model-PHOT*: Model trained on *Photo-CXP*; *Model-RECA*: Recalibrated model trained on CheXpert CXR)

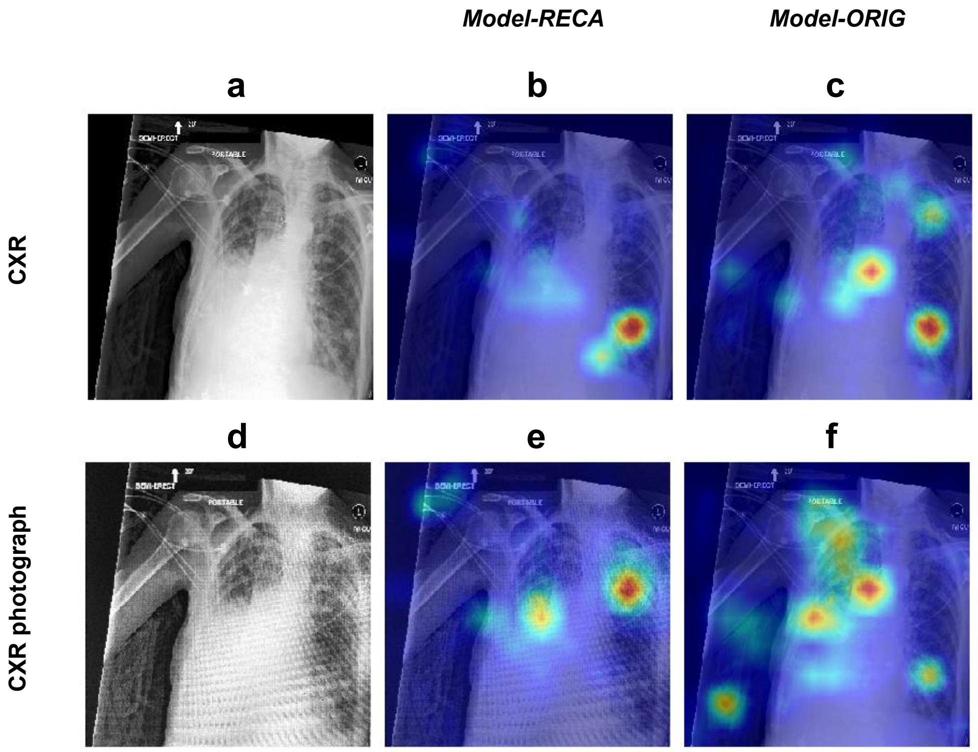

Supplementary Figure 4. An example of the visualization of the diagnostic focus which is influenced by the serious noise. **a** An example CXR is diagnosed as consolidation from the radiology report. **b** and **c** show the diagnostic focus of the recalibrated model (*Model-RECA*) and the uncalibrated model (*Model-ORIG*) tested on the original CXR, respectively. **e** and **f** show the diagnostic focus of the *Model-RECA* and the *Model-ORIG* tested on the corresponding CXR photograph, respectively. The colors from blue to red map the strengths of the contribution of each image location from low to high for predicting consolidation.

Supplementary Tables

Supplementary Table 1. The settings of each dataset

<table>
<tr><th>Datasets</th><th>Smartphones</th><th>Monitors</th><th>Participants</th></tr>
<tr><td>Photo-MMC
(n = 1,759)</td><td rowspan="2">Apple iPhone X
Apple iPhone 6s
Apple iPad 2
Acer Z330
Asus Zenfone 5z
Asus Zenfone 3
Samsung A30
Samsung J7</td><td rowspan="2">MSI GE 40
MSI PS63
Asus VE278
Toshiba Portege R700
Apple MacBook Pro13
Lenovo Yoga 520
Dell SE2417HGX
Samsung SyncMaster 191T plus</td><td rowspan="2">One physician and two non-physicians</td></tr>
<tr><td>Photo-CXP
(n = 1,337)</td></tr>
<tr><td>Photo-MED
(n = 1,337)</td><td>Google pixel
Apple iPhone8
Apple iPhone 6s plus
Apple iPhone 7 plus
Asus Zenfone 5z,
iPhone 6s
Apple iPhone XS
Asus ZenFone 3
Apple iPhone XR</td><td>Dell XPS13
Apple MacBook Air
Apple MacBook Pro 13
Apple MacBook Pro 15
Acer vg270k
Acer swift 3
BenQ ew2775zh</td><td>Nine medical residents</td></tr>
<tr><td>Photo-DEV
(n = 2,020)</td><td>Apple iPhone 6s
Acer Z330
Asus Zenfone 5z</td><td>MSI GE 40,
Dell SE2417HGX
Asus VE278</td><td>One physician</td></tr>
</table>

Supplementary Table 2. Results using MIMIC-based models in internal validation.

		Comparison reference		*Model-ORIG*		*Model-RECA*	
**No finding**	**AUC**	0.8455	± 0.0097	0.8232	± 0.0151	0.8187	± 0.0104
	**Sen.**	78.9	± 2.12	76.06	± 3.76	76.19	± 2.61
	**Spe.**	76.04	± 1.96	77.23	± 3.98	74.94	± 2.74
	**f1**	0.7186	± 0.0134	0.5107	± 0.0315	0.6966	± 0.0133
	**Acc.**	77.1	± 1.08	77.04	± 2.99	75.4	± 1.25
**Enlarg cardiomediastinum**	**AUC**	0.813	± 0.0354	0.667	± 0.038	0.7931	± 0.0333
	**Sen.**	72.23	± 6.11	59.31	± 5.95	72.57	± 5.62
	**Spe.**	82.7	± 1.77	69.23	± 6.07	74	± 2.97
	**f1**	0.188	± 0.0278	0.1662	± 0.0298	0.1363	± 0.0216
	**Acc.**	82.4	± 1.73	68.72	± 5.64	73.96	± 2.88
**Cardiomegaly**	**AUC**	0.814	± 0.0108	0.7704	± 0.0217	0.7986	± 0.0112
	**Sen.**	76.77	± 2.86	71.64	± 4.63	78.76	± 3.22
	**Spe.**	71.57	± 2.41	72.76	± 4.43	68.03	± 2.51
	**f1**	0.5329	± 0.0194	0.3414	± 0.0302	0.5181	± 0.0178
	**Acc.**	72.63	± 1.64	72.65	± 3.72	70.21	± 1.59
**Airspace opacity**	**AUC**	0.73	± 0.013	0.7063	± 0.0142	0.6938	± 0.0142
	**Sen.**	70.38	± 2.94	69.13	± 4.84	67.37	± 2.83
	**Spe.**	63.89	± 2.54	62.69	± 4.16	61.35	± 2.27
	**f1**	0.4606	± 0.0179	0.6256	± 0.0203	0.4317	± 0.0184
	**Acc.**	65.26	± 1.72	65.39	± 1.32	62.62	± 1.6
**Lung lesion**	**AUC**	0.7404	± 0.033	0.7155	± 0.0359	0.6646	± 0.0373
	**Sen.**	65.61	± 6.46	66.65	± 6.39	55.31	± 6.52
	**Spe.**	69.31	± 6.53	68.77	± 6.53	71.76	± 3.78
	**f1**	0.1199	± 0.0227	0.1517	± 0.028	0.1079	± 0.0188
	**Acc.**	69.2	± 6.22	68.69	± 6.12	71.25	± 3.63
**Edema**	**AUC**	0.8929	± 0.012	0.7863	± 0.0144	0.8775	± 0.0122
	**Sen.**	82.01	± 2.59	73.16	± 3.39	78.68	± 2.93
	**Spe.**	82.55	± 2.28	71.88	± 3.47	81.07	± 2.1
	**f1**	0.4785	± 0.0344	0.5544	± 0.0217	0.4454	± 0.0319
	**Acc.**	82.5	± 2	72.19	± 2.2	80.84	± 1.86
**Consolidation**	**AUC**	0.8549	± 0.0177	0.7248	± 0.0299	0.8097	± 0.0217
	**Sen.**	82.13	± 3.75	67.42	± 6.1	73.37	± 4.33
	**Spe.**	75.02	± 3.19	65.87	± 5.89	74.38	± 2.9
	**f1**	0.237	± 0.0282	0.1431	± 0.0222	0.2101	± 0.0256
	**Acc.**	75.35	± 2.96	65.93	± 5.51	74.33	± 2.75

**Pneumonia**	**AUC**	0.7627	± 0.0223	0.6278	± 0.06	0.6909	± 0.0236
	**Sen.**	71.84	± 4.43	47.96	± 9.72	64.23	± 7.11
	**Spe.**	68.21	± 3.52	67.54	± 13.21	61.91	± 7.21
	**f1**	0.2217	± 0.0228	0.055	± 0.0212	0.1755	± 0.0192
	**Acc.**	68.44	± 3.19	67.18	± 12.89	62.05	± 6.4
**Atelectasis**	**AUC**	0.807	± 0.0117	0.6383	± 0.0187	0.7868	± 0.0123
	**Sen.**	80.16	± 2.86	66.96	± 4.39	75.68	± 2.99
	**Spe.**	70.85	± 2.26	57.42	± 3.66	69.46	± 2.65
	**f1**	0.5272	± 0.0193	0.3498	± 0.0197	0.4955	± 0.0198
	**Acc.**	72.62	± 1.56	58.99	± 2.61	70.65	± 1.85
**Pneumothorax**	**AUC**	0.8669	± 0.0226	0.7587	± 0.03	0.8408	± 0.023
	**Sen.**	79.76	± 4.11	66.78	± 4.91	71.8	± 5
	**Spe.**	81.43	± 2.9	75.57	± 3.95	79.14	± 5.88
	**f1**	0.24	± 0.0394	0.2192	± 0.0341	0.2054	± 0.044
	**Acc.**	81.37	± 2.79	75.12	± 3.73	78.87	± 5.6
**Pleural effusion**	**AUC**	0.91	± 0.0072	0.8686	± 0.0105	0.8957	± 0.0077
	**Sen.**	85.71	± 1.82	77.81	± 2.44	84.59	± 2.15
	**Spe.**	82.11	± 1.4	79.74	± 2.47	79.7	± 1.62
	**f1**	0.6831	± 0.0181	0.681	± 0.0186	0.6543	± 0.0181
	**Acc.**	82.89	± 1.08	79.19	± 1.52	80.76	± 1.15
**Pleural(other)**	**AUC**	0.8256	± 0.0391	0.5024	± 0.107	0.8678	± 0.0303
	**Sen.**	70.08	± 6.92	36.34	± 17.09	77.35	± 5.4
	**Spe.**	81.18	± 4.11	55.9	± 20.81	81.41	± 4.97
	**f1**	0.1181	± 0.0297	0.0131	± 0.0113	0.1337	± 0.0379
	**Acc.**	80.99	± 4.05	55.78	± 20.61	81.34	± 4.87
**Fracture**	**AUC**	0.645	± 0.0629	0.6202	± 0.0424	0.5514	± 0.0599
	**Sen.**	60.74	± 8.94	50.54	± 6.57	48.52	± 9.65
	**Spe.**	68.4	± 7.91	69.84	± 7.52	62.48	± 8.08
	**f1**	0.0604	± 0.0184	0.1221	± 0.0283	0.0403	± 0.0117
	**Acc.**	68.28	± 7.74	69.05	± 7.12	62.26	± 7.9
**Support devices**	**AUC**	0.885	± 0.0088	0.7668	± 0.0127	0.8467	± 0.01
	**Sen.**	79.7	± 2.12	70.53	± 2.59	78.34	± 2.29
	**Spe.**	82.78	± 2.11	72.17	± 2.6	76.55	± 2.05
	**f1**	0.6957	± 0.0188	0.7022	± 0.0146	0.6377	± 0.0172
	**Acc.**	81.99	± 1.41	71.38	± 1.17	77.01	± 1.32

Note: AUC: AUROC; Sen.: Sensitivity; Spe.: Specificity; f1: fi-score; Acc: Accuracy.

Supplementary Table 3. Results using MIMIC-based models in external validation.

		Com. Reference	*Model-ORIG*	*Model-TRNS*	*Model-PHOT*	*Model-RECA*
**No finding**	**AUC**	0.838 ± 0.0136	0.7329 ± 0.0177	0.7915 ± 0.0165	0.6902 ± 0.0211	0.8052 ± 0.0156
	**Sen.**	79.48 ± 3.01	74.51 ± 4.06	73.85 ± 3.57	66.19 ± 3.52	79.98 ± 2.83
	**Spe.**	73.35 ± 2.45	62.34 ± 3.37	72.24 ± 3.44	65.91 ± 2.88	70.59 ± 1.97
	**f1**	0.4924 ± 0.0248	0.3951 ± 0.0219	0.4575 ± 0.026	0.3787 ± 0.0231	0.4728 ± 0.0234
	**Acc.**	74.31 ± 1.89	64.25 ± 2.48	72.49 ± 2.6	65.95 ± 2.25	72.06 ± 1.58
**Enlarg cardiomediastinum**	**AUC**	0.6712 ± 0.0356	0.5977 ± 0.0367	0.5977 ± 0.0345	0.5688 ± 0.0359	0.6781 ± 0.0358
	**Sen.**	56.61 ± 6.47	51.77 ± 9.01	61.17 ± 9.7	55.68 ± 6.42	63 ± 5.39
	**Spe.**	71.26 ± 8.13	63.11 ± 10.83	53.32 ± 9.48	60.69 ± 5.74	69.72 ± 3.32
	**f1**	0.1699 ± 0.0326	0.1276 ± 0.0229	0.1208 ± 0.0173	0.1273 ± 0.0193	0.1755 ± 0.0261
	**Acc.**	70.5 ± 7.52	62.53 ± 9.9	53.73 ± 8.57	60.43 ± 5.29	69.38 ± 3.12
**Cardiomegaly**	**AUC**	0.8173 ± 0.0193	0.7067 ± 0.0236	0.7385 ± 0.0229	0.6108 ± 0.0247	0.7971 ± 0.0193
	**Sen.**	73.64 ± 3.62	65.36 ± 5.32	66.9 ± 4.15	59.39 ± 7.19	75.5 ± 3.66
	**Spe.**	76.47 ± 2.55	65.44 ± 5.37	71.1 ± 3.41	59.37 ± 7.51	72.6 ± 2.94
	**f1**	0.3788 ± 0.0309	0.2722 ± 0.0254	0.3105 ± 0.0275	0.2243 ± 0.0216	0.3545 ± 0.0283
	**Acc.**	76.19 ± 2.27	65.43 ± 4.49	70.68 ± 2.96	59.36 ± 6.2	72.89 ± 2.54
**Airspace opacity**	**AUC**	0.6907 ± 0.0148	0.634 ± 0.0152	0.6821 ± 0.0142	0.5848 ± 0.016	0.6914 ± 0.0146
	**Sen.**	69.09 ± 4.62	63.28 ± 3.09	65.07 ± 2.97	52.62 ± 3.19	71.45 ± 3.15
	**Spe.**	59.58 ± 3.95	58.59 ± 2.67	62.25 ± 2.8	59.48 ± 3.05	60.74 ± 2.64
	**f1**	0.6134 ± 0.0202	0.5734 ± 0.018	0.5985 ± 0.0173	0.5039 ± 0.0196	0.6327 ± 0.0171
	**Acc.**	63.57 ± 1.33	60.55 ± 1.32	63.43 ± 1.34	56.6 ± 1.43	65.24 ± 1.27
**Lung lesion**	**AUC**	0.7103 ± 0.0387	0.6678 ± 0.0406	0.6402 ± 0.0429	0.5126 ± 0.0416	0.6291 ± 0.0393
	**Sen.**	59.06 ± 6.86	59.47 ± 6.6	60.51 ± 6.46	45.02 ± 8.52	56.66 ± 7.43
	**Spe.**	73.53 ± 8.75	71.11 ± 7.07	65.3 ± 5.31	60.37 ± 9.11	62.28 ± 7.42
	**f1**	0.1601 ± 0.0404	0.1459 ± 0.0291	0.1258 ± 0.0219	0.085 ± 0.0164	0.1109 ± 0.0205
	**Acc.**	72.93 ± 8.25	70.63 ± 6.67	65.1 ± 5.01	59.74 ± 8.51	62.05 ± 6.94
**Edema**	**AUC**	0.7796 ± 0.0144	0.6811 ± 0.0168	0.7187 ± 0.016	0.6226 ± 0.0182	0.7641 ± 0.0155
	**Sen.**	72.26 ± 2.78	64.84 ± 3.65	68.63 ± 4.24	57.64 ± 4.05	71.37 ± 2.62
	**Spe.**	70.78 ± 2.47	61.13 ± 3.41	63.94 ± 4.07	61.69 ± 4.25	70.66 ± 2.18
	**f1**	0.5418 ± 0.0217	0.4463 ± 0.0206	0.4812 ± 0.0206	0.4094 ± 0.0206	0.5361 ± 0.0219
	**Acc.**	71.13 ± 1.72	62.01 ± 2.18	65.04 ± 2.44	60.74 ± 2.64	70.82 ± 1.62
**Consolidation**	**AUC**	0.7564 ± 0.0302	0.675 ± 0.0329	0.7368 ± 0.0292	0.6112 ± 0.0369	0.7425 ± 0.0288
	**Sen.**	67.19 ± 5.55	66.03 ± 8.2	75.58 ± 5.07	63.58 ± 7.54	71.15 ± 5.2
	**Spe.**	70.47 ± 4.54	56.44 ± 7.81	63.21 ± 2.92	54.5 ± 6.21	68.34 ± 3.41
	**f1**	0.1606 ± 0.0268	0.115 ± 0.0209	0.1484 ± 0.0206	0.1056 ± 0.0159	0.159 ± 0.023
	**Acc.**	70.34 ± 4.31	56.85 ± 7.25	63.73 ± 2.76	54.89 ± 5.75	68.46 ± 3.24

**Pneumonia**	**AUC**	0.6369 ± 0.0572	0.6746 ± 0.0538	0.6971 ± 0.064	0.5362 ± 0.0568	0.6068 ± 0.0607
	**Sen.**	56.8 ± 11	66.12 ± 9.04	58.63 ± 8.49	53.63 ± 10.13	54.52 ± 9.25
	**Spe.**	60.8 ± 11.86	63.53 ± 5.23	74.63 ± 8.01	51.81 ± 9.88	60.9 ± 9.39
	**f1**	0.052 ± 0.016	0.0622 ± 0.0157	0.0804 ± 0.0247	0.0392 ± 0.0101	0.0494 ± 0.0147
	**Acc.**	60.73 ± 11.51	63.59 ± 5.09	74.34 ± 7.86	51.84 ± 9.59	60.78 ± 9.15
**Atelectasis**	**AUC**	0.632 ± 0.0194	0.6115 ± 0.0204	0.6235 ± 0.0187	0.5329 ± 0.0205	0.6221 ± 0.0193
	**Sen.**	63.33 ± 4.33	57.99 ± 6.43	66.84 ± 3.82	54.4 ± 4.59	59.56 ± 3.44
	**Spe.**	58.7 ± 3.78	58.39 ± 6.45	54.69 ± 2.92	53.17 ± 4.18	61.4 ± 2.74
	**f1**	0.3399 ± 0.0207	0.3143 ± 0.0193	0.337 ± 0.0194	0.2775 ± 0.019	0.3353 ± 0.0211
	**Acc.**	59.47 ± 2.75	58.33 ± 4.49	56.69 ± 2.16	53.37 ± 3.03	61.09 ± 2.14
**Pneumothorax**	**AUC**	0.7266 ± 0.0343	0.5822 ± 0.038	0.6367 ± 0.0408	0.5216 ± 0.0316	0.7119 ± 0.0341
	**Sen.**	67.23 ± 5.98	53.63 ± 5.74	57.99 ± 6.36	52.5 ± 10.34	64.47 ± 9.57
	**Spe.**	68.11 ± 6.02	62.89 ± 5.04	69.02 ± 5.86	51.04 ± 10.66	66.99 ± 11.88
	**f1**	0.1809 ± 0.0279	0.1293 ± 0.0206	0.1618 ± 0.0278	0.1002 ± 0.0141	0.1767 ± 0.0401
	**Acc.**	68.07 ± 5.54	62.41 ± 4.7	68.45 ± 5.43	51.12 ± 9.65	66.86 ± 10.83
**Pleural effusion**	**AUC**	0.8891 ± 0.0096	0.7781 ± 0.0133	0.8318 ± 0.0121	0.6391 ± 0.0165	0.857 ± 0.0111
	**Sen.**	82.13 ± 2.03	73.84 ± 2.96	78.77 ± 2.15	62.23 ± 3.55	77.74 ± 2.62
	**Spe.**	81.06 ± 1.91	67.08 ± 2.47	73.94 ± 1.54	59.61 ± 3.45	78.3 ± 2.5
	**f1**	0.7155 ± 0.0174	0.576 ± 0.0181	0.6454 ± 0.0187	0.4723 ± 0.0191	0.6699 ± 0.0184
	**Acc.**	81.36 ± 1.24	69.01 ± 1.47	75.32 ± 1.22	60.36 ± 1.93	78.14 ± 1.51
**Pleural(other)**	**AUC**	0.6357 ± 0.0945	0.6675 ± 0.1064	0.5851 ± 0.1082	0.6925 ± 0.0704	0.545 ± 0.0827
	**Sen.**	57.24 ± 13.6	52.36 ± 16.35	41.99 ± 16.17	58.46 ± 15.09	54.63 ± 15.99
	**Spe.**	58.97 ± 8.39	68.34 ± 14.26	64.96 ± 14.77	68.22 ± 5.41	48.58 ± 13.36
	**f1**	0.0201 ± 0.0136	0.0298 ± 0.0236	0.0184 ± 0.0115	0.0249 ± 0.0106	0.0149 ± 0.007
	**Acc.**	58.97 ± 8.32	68.24 ± 14.13	64.81 ± 14.64	68.16 ± 5.38	48.63 ± 13.22
**Fracture**	**AUC**	0.5849 ± 0.0402	0.576 ± 0.0414	0.5731 ± 0.0388	0.5614 ± 0.0454	0.5344 ± 0.0316
	**Sen.**	54.23 ± 10.81	53.02 ± 6.97	55.69 ± 7.49	58.52 ± 7.13	60.32 ± 6.31
	**Spe.**	57.42 ± 12.58	59.86 ± 6.63	56.1 ± 7.07	57.35 ± 4.79	50.33 ± 3.48
	**f1**	0.0967 ± 0.0179	0.0984 ± 0.0188	0.095 ± 0.0164	0.102 ± 0.0188	0.0915 ± 0.015
	**Acc.**	57.29 ± 11.67	59.58 ± 6.25	56.08 ± 6.6	57.4 ± 4.48	50.74 ± 3.28
**Support devices**	**AUC**	0.7602 ± 0.0127	0.6264 ± 0.0152	0.6825 ± 0.0144	0.5559 ± 0.0151	0.6645 ± 0.0146
	**Sen.**	71.55 ± 3.1	58.33 ± 4.5	62.39 ± 3.17	50.41 ± 3.06	62.04 ± 2.73
	**Spe.**	69.38 ± 3.02	60.21 ± 4.66	67.07 ± 3.34	59.24 ± 3.28	65.14 ± 2.86
	**f1**	0.6982 ± 0.016	0.5778 ± 0.0224	0.6291 ± 0.0171	0.5172 ± 0.0191	0.6202 ± 0.0168
	**Acc.**	70.42 ± 1.23	59.31 ± 1.35	64.82 ± 1.28	55.01 ± 1.31	63.65 ± 1.3

Note: AUC: AUROC; Sen.: Sensitivity; Spe.: Specificity; f1: fi-score; Acc: Accuracy.

Supplementary Table 4. Results using MIMIC-based models in end-user scenario.

		Com. Reference		*Model-ORIG*		*Model-TRNS*		*Model-PHOT*		*Model-RECA*	
**No finding**	**AUC**	0.838	± 0.0136	0.7397	± 0.0186	0.8018	± 0.0159	0.6952	± 0.0203	0.8041	± 0.0158
	**Sen.**	79.48	± 3.01	66.56	± 4	75.42	± 3.35	66.07	± 3.78	72.88	± 3.35
	**Spe.**	73.35	± 2.45	69.13	± 4.29	71.96	± 3.09	66.23	± 3.36	72.69	± 3.27
	**f1**	0.4924	± 0.0248	0.401	± 0.027	0.4627	± 0.0259	0.3802	± 0.0237	0.4563	± 0.0274
	**Acc.**	74.31	± 1.89	68.73	± 3.26	72.5	± 2.37	66.21	± 2.58	72.72	± 2.53
**Enlarg cardiomediastinum**	**AUC**	0.6712	± 0.0356	0.679	± 0.033	0.6397	± 0.0381	0.5738	± 0.0381	0.7003	± 0.0335
	**Sen.**	56.61	± 6.47	62.43	± 6.33	55.29	± 6.19	55.21	± 7.36	62.8	± 5.95
	**Spe.**	71.26	± 8.13	63.19	± 6.56	68.13	± 7.38	59.32	± 7.65	70.33	± 6.28
	**f1**	0.1699	± 0.0326	0.1502	± 0.0225	0.1526	± 0.0299	0.1231	± 0.0188	0.1798	± 0.0291
	**Acc.**	70.5	± 7.52	63.16	± 6.03	67.47	± 6.86	59.11	± 7	69.94	± 5.81
**Cardiomegaly**	**AUC**	0.8173	± 0.0193	0.7414	± 0.0224	0.7839	± 0.0206	0.5792	± 0.0255	0.8081	± 0.0206
	**Sen.**	73.64	± 3.62	67.28	± 4.34	71.98	± 3.9	58.87	± 5.23	72.49	± 3.85
	**Spe.**	76.47	± 2.55	67.52	± 4.48	71.08	± 3.11	54.6	± 4.47	73.61	± 3.09
	**f1**	0.3788	± 0.0309	0.2905	± 0.0264	0.33	± 0.0287	0.2048	± 0.0191	0.3508	± 0.031
	**Acc.**	76.19	± 2.27	67.5	± 3.81	71.17	± 2.69	55.02	± 3.72	73.5	± 2.7
**Airspace opacity**	**AUC**	0.6907	± 0.0148	0.6462	± 0.0147	0.6723	± 0.0147	0.5822	± 0.0164	0.6825	± 0.0149
	**Sen.**	69.09	± 4.62	63.19	± 4.21	69.79	± 3.16	57.76	± 3.7	65.91	± 4.32
	**Spe.**	59.58	± 3.95	58.44	± 3.82	57.9	± 2.5	55.76	± 3.72	61.58	± 3.93
	**f1**	0.6134	± 0.0202	0.572	± 0.0202	0.6117	± 0.0182	0.527	± 0.0203	0.6011	± 0.0194
	**Acc.**	63.57	± 1.33	60.43	± 1.36	62.89	± 1.34	56.6	± 1.5	63.39	± 1.34
**Lung lesion**	**AUC**	0.7103	± 0.0387	0.6241	± 0.0394	0.6586	± 0.0389	0.5291	± 0.0352	0.6545	± 0.0362
	**Sen.**	59.06	± 6.86	57.57	± 7.22	64.12	± 8	56.6	± 6.41	57.8	± 7.5
	**Spe.**	73.53	± 8.75	62.09	± 6.44	58.76	± 8.23	53.87	± 4.6	64.4	± 9.19
	**f1**	0.1601	± 0.0404	0.1116	± 0.0199	0.1153	± 0.0193	0.0919	± 0.015	0.1208	± 0.0272
	**Acc.**	72.93	± 8.25	61.91	± 6.04	58.98	± 7.65	53.98	± 4.32	64.13	± 8.61
**Edema**	**AUC**	0.7796	± 0.0144	0.6899	± 0.0161	0.7397	± 0.0158	0.6617	± 0.0172	0.769	± 0.015
	**Sen.**	72.26	± 2.78	64.3	± 5.12	68.32	± 4.15	61.54	± 2.71	70.9	± 2.84
	**Spe.**	70.78	± 2.47	62.87	± 5.36	67.16	± 4.01	66.51	± 1.95	71.26	± 2.46
	**f1**	0.5418	± 0.0217	0.4523	± 0.0205	0.4978	± 0.0212	0.456	± 0.0209	0.5375	± 0.0214
	**Acc.**	71.13	± 1.72	63.21	± 3.14	67.43	± 2.42	65.33	± 1.52	71.17	± 1.7
**Consolidation**	**AUC**	0.7564	± 0.0302	0.6957	± 0.0355	0.7247	± 0.0349	0.6024	± 0.0363	0.7295	± 0.0317
	**Sen.**	67.19	± 5.55	67.88	± 7.78	64.96	± 6.21	57.79	± 6.84	67.29	± 7.65
	**Spe.**	70.47	± 4.54	60.61	± 7.55	68.19	± 6.14	56.26	± 6.27	65.74	± 8.36
	**f1**	0.1606	± 0.0268	0.1282	± 0.0203	0.1476	± 0.0267	0.1001	± 0.0164	0.1444	± 0.0259
	**Acc.**	70.34	± 4.31	60.92	± 6.98	68.06	± 5.76	56.32	± 5.87	65.8	± 7.77

**Pneumonia**	**AUC**	0.6369 ± 0.0572	0.6168 ± 0.0577	0.6308 ± 0.0588	0.5652 ± 0.0647	0.6185 ± 0.0646
	**Sen.**	56.8 ± 11	52.29 ± 9.77	49.73 ± 10.23	44.94 ± 10.57	51.07 ± 10.46
	**Spe.**	60.8 ± 11.86	61.93 ± 10.04	69.33 ± 13.15	66.67 ± 13.68	69.29 ± 12.91
	**f1**	0.052 ± 0.016	0.0491 ± 0.016	0.0598 ± 0.0212	0.0502 ± 0.019	0.0618 ± 0.0232
	**Acc.**	60.73 ± 11.51	61.76 ± 9.78	68.97 ± 12.82	66.27 ± 13.34	68.96 ± 12.57
**Atelectasis**	**AUC**	0.632 ± 0.0194	0.6108 ± 0.0193	0.6028 ± 0.0193	0.5818 ± 0.0211	0.6136 ± 0.0187
	**Sen.**	63.33 ± 4.33	61.63 ± 5.38	60.69 ± 4.27	55.85 ± 5.72	66.43 ± 5.32
	**Spe.**	58.7 ± 3.78	55.36 ± 4.83	55.09 ± 3.5	57.27 ± 5.9	52.79 ± 4.28
	**f1**	0.3399 ± 0.0207	0.3176 ± 0.0191	0.3124 ± 0.0195	0.2999 ± 0.02	0.3272 ± 0.0194
	**Acc.**	59.47 ± 2.75	56.4 ± 3.37	56.01 ± 2.54	57.04 ± 4.19	55.04 ± 2.94
**Pneumothorax**	**AUC**	0.7266 ± 0.0343	0.6737 ± 0.0376	0.648 ± 0.0399	0.576 ± 0.0361	0.7371 ± 0.0323
	**Sen.**	67.23 ± 5.98	60.49 ± 6.07	57.59 ± 6.2	53.9 ± 7.86	70.1 ± 5.97
	**Spe.**	68.11 ± 6.02	65.38 ± 5.81	66.74 ± 5.56	58.66 ± 8.69	66.77 ± 5.8
	**f1**	0.1809 ± 0.0279	0.1537 ± 0.0258	0.152 ± 0.0268	0.1195 ± 0.0197	0.1822 ± 0.0294
	**Acc.**	68.07 ± 5.54	65.12 ± 5.37	66.27 ± 5.18	58.41 ± 7.93	66.95 ± 5.33
**Pleural effusion**	**AUC**	0.8891 ± 0.0096	0.7914 ± 0.0136	0.8487 ± 0.0115	0.6759 ± 0.0159	0.8645 ± 0.0109
	**Sen.**	82.13 ± 2.03	75.81 ± 3.01	77.32 ± 2.15	61.9 ± 3.23	79.76 ± 2.4
	**Spe.**	81.06 ± 1.91	67.57 ± 2.55	79.09 ± 1.6	65.93 ± 3.31	79.01 ± 2.25
	**f1**	0.7155 ± 0.0174	0.5898 ± 0.0182	0.6732 ± 0.019	0.5007 ± 0.0189	0.6866 ± 0.0183
	**Acc.**	81.36 ± 1.24	69.92 ± 1.51	78.59 ± 1.25	64.78 ± 1.93	79.23 ± 1.41
**Pleural(other)**	**AUC**	0.6357 ± 0.0945	0.6352 ± 0.0938	0.7847 ± 0.0454	0.392 ± 0.0747	0.6754 ± 0.0842
	**Sen.**	57.24 ± 13.6	49.53 ± 16	63.92 ± 14.1	44.29 ± 17.85	49.58 ± 15.25
	**Spe.**	58.97 ± 8.39	60.51 ± 17.09	72.53 ± 10.16	38.55 ± 13	63.76 ± 14.28
	**f1**	0.0201 ± 0.0136	0.0234 ± 0.0216	0.0333 ± 0.015	0.0095 ± 0.0047	0.0225 ± 0.0182
	**Acc.**	58.97 ± 8.32	60.45 ± 16.93	72.47 ± 10.05	38.59 ± 12.84	63.67 ± 14.14
**Fracture**	**AUC**	0.5849 ± 0.0402	0.5644 ± 0.0411	0.6063 ± 0.0391	0.5235 ± 0.0408	0.6498 ± 0.0348
	**Sen.**	54.23 ± 10.81	52.79 ± 6.76	54.18 ± 6.17	47.71 ± 6.91	57.94 ± 7.23
	**Spe.**	57.42 ± 12.58	58.87 ± 4.82	68.82 ± 3.8	59.84 ± 5.63	64.03 ± 8.13
	**f1**	0.0967 ± 0.0179	0.0951 ± 0.0168	0.1236 ± 0.0225	0.0884 ± 0.0169	0.1184 ± 0.0223
	**Acc.**	57.29 ± 11.67	58.62 ± 4.55	68.22 ± 3.65	59.34 ± 5.29	63.78 ± 7.61
**Support devices**	**AUC**	0.7602 ± 0.0127	0.6836 ± 0.0145	0.6893 ± 0.0139	0.5415 ± 0.016	0.6789 ± 0.0143
	**Sen.**	71.55 ± 3.1	65.82 ± 2.8	60.56 ± 3.53	55.44 ± 4.95	64.18 ± 3.55
	**Spe.**	69.38 ± 3.02	63.63 ± 2.64	69.24 ± 4.08	52.7 ± 4.73	62.74 ± 3.63
	**f1**	0.6982 ± 0.016	0.6406 ± 0.017	0.6238 ± 0.0175	0.5348 ± 0.0257	0.6265 ± 0.0179
	**Acc.**	70.42 ± 1.23	64.68 ± 1.28	65.09 ± 1.33	54.01 ± 1.36	63.43 ± 1.27

Note: AUC: AUROC; Sen.: Sensitivity; Spe.: Specificity; f1: fi-score; Acc: Accuracy.

Supplementary Table 5. Results using CheXpert-based models in internal validation.

		Comparison reference	*Model-ORIG*	*Model-RECA*
**No finding**	**AUC**	0.8333 ± 0.014	0.6975 ± 0.0192	0.7946 ± 0.0161
	**Sen.**	73.67 ± 3.35	65.28 ± 4.50	71.87 ± 4.06
	**Spe.**	79.8 ± 3.80	65.87 ± 4.53	73.04 ± 4.21
	**f1**	0.5233 ± 0.0345	0.3746 ± 0.0238	0.4546 ± 0.0289
	**Acc.**	78.84 ± 2.96	65.77 ± 3.36	72.86 ± 3.17
**Enlarg cardiomediastinum**	**AUC**	0.7245 ± 0.0302	0.5497 ± 0.034	0.7197 ± 0.0334
	**Sen.**	61.7 ± 5.78	62.26 ± 6.36	63.59 ± 5.47
	**Spe.**	73.46 ± 6.42	48.18 ± 4.82	70.33 ± 5.57
	**f1**	0.1932 ± 0.0323	0.1116 ± 0.0152	0.1816 ± 0.0296
	**Acc.**	72.85 ± 5.93	48.91 ± 4.42	69.98 ± 5.17
**Cardiomegaly**	**AUC**	0.855 ± 0.0178	0.6322 ± 0.0243	0.8108 ± 0.0189
	**Sen.**	78.3 ± 3.89	62.72 ± 7.43	70.49 ± 4.03
	**Spe.**	78.12 ± 3.71	57.05 ± 7.41	74.98 ± 4.35
	**f1**	0.4153 ± 0.0365	0.2262 ± 0.0202	0.3549 ± 0.0357
	**Acc.**	78.14 ± 3.15	57.61 ± 6.06	74.54 ± 3.75
**Airspace opacity**	**AUC**	0.7406 ± 0.0137	0.6652 ± 0.0151	0.7019 ± 0.0142
	**Sen.**	69.12 ± 3.41	60.5 ± 2.92	67.92 ± 4.18
	**Spe.**	68.45 ± 3.33	65.08 ± 2.91	62.03 ± 3.66
	**f1**	0.6494 ± 0.0165	0.5792 ± 0.0178	0.6157 ± 0.0193
	**Acc.**	68.73 ± 1.34	63.16 ± 1.4	64.51 ± 1.3
**Lung lesion**	**AUC**	0.6466 ± 0.0367	0.5877 ± 0.0345	0.6904 ± 0.0319
	**Sen.**	61.76 ± 7.3	57.91 ± 10.39	68.3 ± 5.81
	**Spe.**	58.44 ± 6.68	55.19 ± 10.62	62.61 ± 3.62
	**f1**	0.11 ± 0.0185	0.0973 ± 0.0163	0.1315 ± 0.0204
	**Acc.**	58.57 ± 6.23	55.3 ± 9.83	62.84 ± 3.42
**Edema**	**AUC**	0.8047 ± 0.0133	0.6907 ± 0.0169	0.7368 ± 0.0166
	**Sen.**	76.01 ± 3.33	63.02 ± 3.87	66.49 ± 2.78
	**Spe.**	69.76 ± 3.03	66.17 ± 4.09	69.75 ± 2.51
	**f1**	0.5553 ± 0.0211	0.4628 ± 0.0207	0.5032 ± 0.0219
	**Acc.**	71.23 ± 1.91	65.43 ± 2.56	68.98 ± 1.8
**Consolidation**	**AUC**	0.7594 ± 0.0303	0.61 ± 0.0338	0.7505 ± 0.0266
	**Sen.**	66.52 ± 7.6	63.26 ± 6.43	76.5 ± 5.2
	**Spe.**	69.71 ± 9.22	54.41 ± 4.21	65.08 ± 2.93
	**f1**	0.161 ± 0.0358	0.1047 ± 0.0159	0.1566 ± 0.0216
	**Acc.**	69.58 ± 8.6	54.78 ± 3.9	65.56 ± 2.73

**Pneumonia**	**AUC**	0.7202	± 0.0467	0.5388	± 0.0509	0.6457	± 0.0532
	**Sen.**	66.14	± 8.72	57.78	± 10.53	59.5	± 8.32
	**Spe.**	65.63	± 7.86	50.01	± 7.34	64.12	± 6.64
	**f1**	0.0668	± 0.0177	0.0402	± 0.01	0.0575	± 0.0156
	**Acc.**	65.65	± 7.64	50.15	± 7.11	64.04	± 6.49
**Atelectasis**	**AUC**	0.6348	± 0.0193	0.551	± 0.0212	0.5851	± 0.0196
	**Sen.**	60.6	± 5.99	54.25	± 5.77	60.77	± 6.37
	**Spe.**	59.56	± 5.79	53.36	± 5.9	54.5	± 6.17
	**f1**	0.3315	± 0.0196	0.2775	± 0.0183	0.3103	± 0.0188
	**Acc.**	59.73	± 4.04	53.51	± 4.19	55.53	± 4.28
**Pneumothorax**	**AUC**	0.7398	± 0.0299	0.551	± 0.0344	0.6918	± 0.0342
	**Sen.**	67.83	± 5.31	50.97	± 6.02	65.59	± 5.35
	**Spe.**	68.8	± 4.49	60.98	± 4.37	67.1	± 4.19
	**f1**	0.1842	± 0.0253	0.1178	± 0.0186	0.1715	± 0.0249
	**Acc.**	68.75	± 4.16	60.46	± 4.07	67.03	± 3.91
**Pleural effusion**	**AUC**	0.8831	± 0.0099	0.8017	± 0.0125	0.8536	± 0.011
	**Sen.**	82.52	± 2.08	73.98	± 3.15	77.7	± 2.37
	**Spe.**	80.64	± 1.76	70.94	± 3.02	78.35	± 2.16
	**f1**	0.7144	± 0.0177	0.5996	± 0.0184	0.67	± 0.0186
	**Acc.**	81.18	± 1.2	71.81	± 1.67	78.17	± 1.4
**Pleural(other)**	**AUC**	0.5832	± 0.1172	0.3762	± 0.0805	0.5687	± 0.0837
	**Sen.**	38.83	± 16.07	46.59	± 19.28	42.7	± 16.88
	**Spe.**	75.69	± 10.07	36.26	± 17	59.59	± 14.72
	**f1**	0.0222	± 0.012	0.0093	± 0.0048	0.0147	± 0.0072
	**Acc.**	75.45	± 9.99	36.34	± 16.79	59.48	± 14.55
**Fracture**	**AUC**	0.6986	± 0.0331	0.5709	± 0.0389	0.6673	± 0.0359
	**Sen.**	66.16	± 6.54	58.84	± 6.57	61.47	± 6.65
	**Spe.**	63.41	± 6.34	56.01	± 5.44	63.42	± 6.8
	**f1**	0.1314	± 0.0227	0.0997	± 0.0164	0.1226	± 0.021
	**Acc.**	63.52	± 5.92	56.12	± 5.11	63.34	± 6.36
**Support devices**	**AUC**	0.7884	± 0.0124	0.6116	± 0.0155	0.7079	± 0.0139
	**Sen.**	72	± 2.59	55.33	± 5.91	67.13	± 2.73
	**Spe.**	75.12	± 2.75	59.84	± 6.64	65.02	± 2.55
	**f1**	0.7231	± 0.014	0.5545	± 0.027	0.654	± 0.0165
	**Acc.**	73.62	± 1.17	57.68	± 1.45	66.03	± 1.27

Note: AUC: AUROC; Sen.: Sensitivity; Spe.: Specificity; f1: fi-score; Acc: Accuracy.

Supplementary Table 6. Results using CheXpert-based models in external validation.

		Com. Reference	*Model-ORIG*	*Model-TRNS*	*Model-PHOT*	*Model-RECA*
**No finding**	**AUC**	0.8144 ± 0.0125	0.716 ± 0.0125	0.7295 ± 0.0118	0.6928 ± 0.0128	0.7697 ± 0.0115
	**Sen.**	75.65 ± 1.87	67.04 ± 2.99	69.38 ± 2.75	65 ± 3.62	69.69 ± 2.81
	**Spe.**	75.63 ± 1.81	66.73 ± 2.9	65.11 ± 2.54	65.21 ± 3.59	72.13 ± 2.85
	**f1**	0.6971 ± 0.0148	0.5997 ± 0.0148	0.6069 ± 0.0149	0.5799 ± 0.0153	0.6422 ± 0.0143
	**Acc.**	75.64 ± 1.08	66.84 ± 1.27	66.69 ± 1.19	65.13 ± 1.42	71.22 ± 1.29
**Enlarg cardiomediastinum**	**AUC**	0.597 ± 0.0457	0.6056 ± 0.0381	0.7184 ± 0.0404	0.5928 ± 0.04	0.7022 ± 0.04
	**Sen.**	55.74 ± 6.95	56.29 ± 7.04	63.1 ± 6.26	54.98 ± 6.63	60.07 ± 7.53
	**Spe.**	63.36 ± 4.51	61.67 ± 6.78	73.48 ± 4.14	60.68 ± 5.30	71.53 ± 11.35
	**f1**	0.0789 ± 0.0145	0.077 ± 0.0145	0.1186 ± 0.0233	0.0732 ± 0.01	0.1196 ± 0.05
	**Acc.**	63.15 ± 4.34	61.51 ± 6.5	73.18 ± 4.01	60.52 ± 5.09	71.21 ± 10.90
**Cardiomegaly**	**AUC**	0.7705 ± 0.0123	0.6518 ± 0.0161	0.7212 ± 0.0151	0.6119 ± 0.02	0.7361 ± 0.01
	**Sen.**	73.26 ± 3.18	61.31 ± 3.66	63.76 ± 3.15	56.51 ± 3.24	70.05 ± 3.37
	**Spe.**	67.52 ± 2.88	59.94 ± 3.78	70.44 ± 3.3	62.64 ± 2.88	63.98 ± 3.05
	**f1**	0.4877 ± 0.0186	0.3854 ± 0.0181	0.4564 ± 0.0203	0.3731 ± 0.0188	0.4503 ± 0.0178
	**Acc.**	68.69 ± 1.91	60.22 ± 2.54	69.08 ± 2.3	61.4 ± 2.02	65.22 ± 2.01
**Airspace opacity**	**AUC**	0.6955 ± 0.0139	0.6454 ± 0.0157	0.6394 ± 0.0154	0.6118 ± 0.0166	0.6649 ± 0.0149
	**Sen.**	70.44 ± 3.2	61.95 ± 3.71	60.87 ± 3.62	59.29 ± 3.47	70.35 ± 3.46
	**Spe.**	61.37 ± 2.68	60.99 ± 3.67	60.45 ± 3.6	57 ± 3.35	56.71 ± 2.7
	**f1**	0.447 ± 0.0175	0.4022 ± 0.0183	0.394 ± 0.0177	0.3701 ± 0.0173	0.4231 ± 0.0174
	**Acc.**	63.28 ± 1.76	61.19 ± 2.39	60.54 ± 2.37	57.48 ± 2.24	59.59 ± 1.73
**Lung lesion**	**AUC**	0.674 ± 0.0388	0.586 ± 0.0413	0.6088 ± 0.0402	0.4506 ± 0.038	0.6133 ± 0.042
	**Sen.**	57.22 ± 6.23	52.66 ± 8.31	61.07 ± 6.5	50.11 ± 8.88	57.01 ± 7.25
	**Spe.**	72.48 ± 6.6	61.69 ± 8.94	60.28 ± 5.25	46.74 ± 8.04	62.9 ± 6.97
	**f1**	0.1162 ± 0.0235	0.08 ± 0.0152	0.0883 ± 0.0149	0.0555 ± 0.009	0.0887 ± 0.017
	**Acc.**	72 ± 6.34	61.42 ± 8.48	60.3 ± 5.01	46.85 ± 7.58	62.72 ± 6.63
**Edema**	**AUC**	0.8698 ± 0.0134	0.7803 ± 0.016	0.8209 ± 0.0165	0.7365 ± 0.0206	0.8173 ± 0.0167
	**Sen.**	81.62 ± 3.37	74.64 ± 3.48	71.92 ± 3.64	68.6 ± 3.55	72.92 ± 3.28
	**Spe.**	77.97 ± 3.22	70.56 ± 3.07	78.43 ± 4.48	70.74 ± 2.92	76.65 ± 1.72
	**f1**	0.4249 ± 0.0318	0.3344 ± 0.0232	0.3905 ± 0.0391	0.3128 ± 0.0255	0.375 ± 0.0256
	**Acc.**	78.32 ± 2.7	70.96 ± 2.6	77.8 ± 3.85	70.53 ± 2.55	76.29 ± 1.57
**Consolidation**	**AUC**	0.7837 ± 0.0203	0.6574 ± 0.0284	0.6839 ± 0.0277	0.5628 ± 0.0322	0.7508 ± 0.0237
	**Sen.**	80.78 ± 4.65	68.15 ± 6	61.95 ± 6.87	60.58 ± 5.82	73.5 ± 5.37
	**Spe.**	67.59 ± 3.43	56.86 ± 4.78	64.64 ± 7.42	53.67 ± 3.96	65.87 ± 4.64
	**f1**	0.191 ± 0.0215	0.1294 ± 0.016	0.1412 ± 0.0208	0.1088 ± 0.0139	0.1686 ± 0.0205
	**Acc.**	68.2 ± 3.14	57.38 ± 4.39	64.52 ± 6.85	54 ± 3.66	66.22 ± 4.27

**Pneumonia**	**AUC**	0.5945 ± 0.031	0.5935 ± 0.0282	0.5132 ± 0.0304	0.5091 ± 0.028	0.5806 ± 0.0294
	**Sen.**	49.64 ± 4.85	52.74 ± 5.62	51.09 ± 8.23	44.62 ± 6.46	52.89 ± 5.82
	**Spe.**	67.53 ± 3.33	62.54 ± 6.47	54.42 ± 9.56	62.34 ± 7.44	58.68 ± 5.55
	**f1**	0.1558 ± 0.0195	0.1486 ± 0.0198	0.1229 ± 0.015	0.1262 ± 0.0165	0.1371 ± 0.0166
	**Acc.**	66.42 ± 3.09	61.93 ± 5.85	54.21 ± 8.54	61.24 ± 6.69	58.32 ± 4.98
**Atelectasis**	**AUC**	0.7656 ± 0.0125	0.6869 ± 0.0158	0.6275 ± 0.0155	0.536 ± 0.0177	0.7275 ± 0.0142
	**Sen.**	76.78 ± 2.77	64.04 ± 3.79	66.65 ± 3.45	53.79 ± 3.71	71.02 ± 3.25
	**Spe.**	66.12 ± 2.16	62.88 ± 3.57	55.76 ± 2.75	53.76 ± 3.23	64.04 ± 2.85
	**f1**	0.4786 ± 0.0184	0.398 ± 0.0192	0.3756 ± 0.0172	0.3068 ± 0.0173	0.4385 ± 0.0187
	**Acc.**	68.15 ± 1.53	63.11 ± 2.44	57.84 ± 1.9	53.77 ± 2.23	65.37 ± 1.99
**Pneumothorax**	**AUC**	0.6661 ± 0.0347	0.4898 ± 0.032	0.673 ± 0.034	0.5518 ± 0.0395	0.7042 ± 0.034
	**Sen.**	58.75 ± 7.33	51.23 ± 9.87	61.01 ± 6.45	51.98 ± 6.46	69.95 ± 6.85
	**Spe.**	64.71 ± 8.87	50.67 ± 10.19	64.3 ± 6.71	61.83 ± 1.68	65.42 ± 6.51
	**f1**	0.1102 ± 0.0208	0.0705 ± 0.011	0.112 ± 0.0206	0.0892 ± 0.015	0.1309 ± 0.0228
	**Acc.**	64.49 ± 8.36	50.68 ± 9.51	64.18 ± 6.34	61.47 ± 1.61	65.59 ± 6.11
**Pleural effusion**	**AUC**	0.8943 ± 0.0087	0.8301 ± 0.0107	0.8313 ± 0.0113	0.7203 ± 0.0141	0.8652 ± 0.0091
	**Sen.**	84.7 ± 2	76.73 ± 3.09	79.04 ± 2.4	69.9 ± 4.85	81.14 ± 2.28
	**Spe.**	80.25 ± 1.71	73.5 ± 3.01	74.26 ± 2.01	64.1 ± 4.47	76.94 ± 2
	**f1**	0.66 ± 0.0191	0.5617 ± 0.0198	0.5793 ± 0.0182	0.4648 ± 0.0175	0.612 ± 0.0189
	**Acc.**	81.21 ± 1.26	74.2 ± 1.94	75.29 ± 1.4	65.35 ± 2.66	77.84 ± 1.39
**Pleural(other)**	**AUC**	0.751 ± 0.0387	0.6055 ± 0.0457	0.6073 ± 0.0532	0.5322 ± 0.0538	0.7612 ± 0.0327
	**Sen.**	69.87 ± 7.11	67.39 ± 8.08	60.07 ± 8.47	51.94 ± 8.72	69.82 ± 6.79
	**Spe.**	72.4 ± 5.13	54.27 ± 3.72	57.6 ± 7.41	55.76 ± 8.54	67.81 ± 6.24
	**f1**	0.0837 ± 0.0206	0.0497 ± 0.0108	0.0484 ± 0.0116	0.0403 ± 0.0098	0.0724 ± 0.0165
	**Acc.**	72.36 ± 5.02	54.51 ± 3.62	57.65 ± 7.21	55.7 ± 8.31	67.84 ± 6.08
**Fracture**	**AUC**	0.5977 ± 0.0458	0.6161 ± 0.048	0.5501 ± 0.0556	0.5904 ± 0.0509	0.5279 ± 0.0526
	**Sen.**	60.27 ± 8.02	64.16 ± 11.03	53.69 ± 9.5	55.94 ± 10.45	54.28 ± 11.24
	**Spe.**	59.61 ± 5.72	54.94 ± 10.34	53.6 ± 9.77	55.88 ± 11.09	49.97 ± 11.6
	**f1**	0.0458 ± 0.0107	0.0447 ± 0.0109	0.0366 ± 0.0108	0.04 ± 0.0104	0.0339 ± 0.008
	**Acc.**	59.62 ± 5.6	55.09 ± 10.04	53.6 ± 9.53	55.88 ± 10.8	50.03 ± 11.28
**Support devices**	**AUC**	0.8145 ± 0.012	0.688 ± 0.0141	0.732 ± 0.0135	0.5568 ± 0.0157	0.7752 ± 0.0119
	**Sen.**	78.49 ± 2.26	65.9 ± 2.56	67.78 ± 3.67	53.6 ± 4.04	72.56 ± 2.96
	**Spe.**	75.69 ± 2.15	63.52 ± 2.19	66.74 ± 3.71	55.9 ± 4.23	70.34 ± 2.66
	**f1**	0.6321 ± 0.0175	0.4868 ± 0.0178	0.5148 ± 0.0177	0.3821 ± 0.0167	0.5629 ± 0.0172
	**Acc.**	76.41 ± 1.4	64.14 ± 1.47	67.01 ± 2.1	55.3 ± 2.39	70.92 ± 1.57

Note: AUC: AUROC; Sen.: Sensitivity; Spe.: Specificity; f1: fi-score; Acc: Accuracy.

Supplementary Table 7. Comparison between different hyper-parameter selection methods.

		Random selection	Manual selection	Similarity comparison
**No finding**	**AUROC**	0.8259 ± 0.0145	0.8213 ± 0.0145	0.7946 ± 0.02
	**Sensitivity**	76.07 ± 3.29	72.75 ± 3.64	71.87 ± 4.06
	**Specificity**	74.19 ± 3.32	76.36 ± 3.65	73.04 ± 4.21
	**f1-score**	0.4838 ± 0.0284	0.4859 ± 0.029	0.4546 ± 0.03
	**Accuracy**	74.49 ± 2.55	75.79 ± 2.77	72.86 ± 3.17
**Enlarg cardiomediastinum**	**AUROC**	0.6613 ± 0.0347	0.6626 ± 0.0327	0.7197 ± 0.03
	**Sensitivity**	61.8 ± 6.7	65.41 ± 6.01	63.59 ± 5.47
	**Specificity**	63.26 ± 7.01	62.24 ± 4.79	70.33 ± 5.57
	**f1-score**	0.1499 ± 0.0263	0.1526 ± 0.0211	0.1816 ± 0.03
	**Accuracy**	63.19 ± 6.44	62.4 ± 4.39	69.98 ± 5.17
**Cardiomegaly**	**AUROC**	0.8207 ± 0.0192	0.8085 ± 0.0198	0.8108 ± 0.02
	**Sensitivity**	74.79 ± 3.86	73.99 ± 4.19	70.49 ± 4.03
	**Specificity**	75.97 ± 3.74	71.95 ± 3.8	74.98 ± 4.35
	**f1-score**	0.3802 ± 0.0348	0.3444 ± 0.0301	0.3549 ± 0.0357
	**Accuracy**	75.85 ± 3.21	72.16 ± 3.22	74.54 ± 3.75
**Airspace opacity**	**AUROC**	0.6929 ± 0.0143	0.6838 ± 0.0147	0.7019 ± 0.0142
	**Sensitivity**	69.01 ± 3.62	66.04 ± 4.48	67.92 ± 4.18
	**Specificity**	59.26 ± 3.11	60.26 ± 3.8	62.03 ± 3.66
	**f1-score**	0.612 ± 0.0183	0.5969 ± 0.0206	0.6157 ± 0.0193
	**Accuracy**	63.35 ± 1.27	62.69 ± 1.31	64.51 ± 1.3
**Lung lesion**	**AUROC**	0.6955 ± 0.0337	0.6837 ± 0.0337	0.6904 ± 0.0319
	**Sensitivity**	60.13 ± 5.92	62.73 ± 6.07	68.3 ± 5.81
	**Specificity**	70.93 ± 4.4	65.73 ± 5.83	62.61 ± 3.62
	**f1-score**	0.1443 ± 0.0242	0.1318 ± 0.0229	0.1315 ± 0.0204
	**Accuracy**	70.49 ± 4.18	65.6 ± 5.48	62.84 ± 3.42
**Edema**	**AUROC**	0.7788 ± 0.0153	0.7746 ± 0.0151	0.7368 ± 0.0166
	**Sensitivity**	69.36 ± 3.14	72.56 ± 2.68	66.49 ± 2.78
	**Specificity**	74.8 ± 3.1	71.14 ± 2.25	69.75 ± 2.51
	**f1-score**	0.5533 ± 0.0226	0.5458 ± 0.0216	0.5032 ± 0.0219
	**Accuracy**	73.52 ± 2.04	71.47 ± 1.61	68.98 ± 1.8
**Consolidation**	**AUROC**	0.7447 ± 0.0304	0.721 ± 0.0328	0.7505 ± 0.0266
	**Sensitivity**	70.89 ± 5.86	67.18 ± 6.33	76.5 ± 5.2
	**Specificity**	66.02 ± 5.21	65.65 ± 6	65.08 ± 2.93
	**f1-score**	0.1507 ± 0.0246	0.1423 ± 0.0236	0.1566 ± 0.0216
	**Accuracy**	66.23 ± 4.86	65.72 ± 5.61	65.56 ± 2.73

**Pneumonia**	**AUROC**	0.6273	± 0.0607	0.6048	± 0.0687	0.6457	± 0.0532
	**Sensitivity**	57.77	± 9.02	48.85	± 9.29	59.5	± 8.32
	**Specificity**	60.69	± 6.72	71.81	± 10.75	64.12	± 6.64
	**f1-score**	0.0517	± 0.0163	0.0631	± 0.0226	0.0575	± 0.0156
	**Accuracy**	60.64	± 6.58	71.4	± 10.5	64.04	± 6.49
**Atelectasis**	**AUROC**	0.6082	± 0.0198	0.6231	± 0.0184	0.5851	± 0.0196
	**Sensitivity**	62.53	± 4.98	66.37	± 4.53	60.77	± 6.37
	**Specificity**	54.04	± 4.23	54.39	± 3.57	54.5	± 6.17
	**f1-score**	0.3161	± 0.0198	0.3337	± 0.0189	0.3103	± 0.0188
	**Accuracy**	55.45	± 2.98	56.37	± 2.5	55.53	± 4.28
**Pneumothorax**	**AUROC**	0.6991	± 0.035	0.6984	± 0.0362	0.6918	± 0.0342
	**Sensitivity**	66.19	± 6.77	68.25	± 5.99	65.59	± 5.35
	**Specificity**	63.8	± 7.21	62.3	± 5.67	67.1	± 4.19
	**f1-score**	0.1618	± 0.0255	0.16	± 0.0237	0.1715	± 0.0249
	**Accuracy**	63.93	± 6.6	62.61	± 5.2	67.03	± 3.91
**Pleural effusion**	**AUROC**	0.8636	± 0.0107	0.8557	± 0.0111	0.8536	± 0.011
	**Sensitivity**	78.13	± 2.33	76.04	± 2.43	77.7	± 2.37
	**Specificity**	78.63	± 2.16	79.48	± 2.4	78.35	± 2.16
	**f1-score**	0.6745	± 0.0187	0.6687	± 0.0196	0.67	± 0.0186
	**Accuracy**	78.49	± 1.42	78.5	± 1.52	78.17	± 1.4
**Pleural(other)**	**AUROC**	0.7435	± 0.0609	0.6589	± 0.0765	0.5687	± 0.0837
	**Sensitivity**	60.67	± 14.97	52.29	± 13.57	42.7	± 16.88
	**Specificity**	64.74	± 11.98	61.07	± 8.92	59.59	± 14.72
	**f1-score**	0.0246	± 0.0112	0.0185	± 0.0082	0.0147	± 0.0072
	**Accuracy**	64.72	± 11.85	61.02	± 8.86	59.48	± 14.55
**Fracture**	**AUROC**	0.6143	± 0.0418	0.5178	± 0.04	0.6673	± 0.0359
	**Sensitivity**	53.14	± 9.11	52.49	± 8.66	61.47	± 6.65
	**Specificity**	62.94	± 11.28	54.36	± 9.05	63.42	± 6.8
	**f1-score**	0.108	± 0.0231	0.0869	± 0.015	0.1226	± 0.021
	**Accuracy**	62.53	± 10.52	54.29	± 8.43	63.34	± 6.36
**Support devices**	**AUROC**	0.6778	± 0.0143	0.6806	± 0.0143	0.7079	± 0.0139
	**Sensitivity**	64.3	± 4.62	61.7	± 3.34	67.13	± 2.73
	**Specificity**	62.85	± 4.49	68.13	± 3.88	65.02	± 2.55
	**f1-score**	0.6274	± 0.0212	0.628	± 0.0164	0.654	± 0.0165
	**Accuracy**	63.54	± 1.28	65.05	± 1.32	66.03	± 1.27

Supplementary Results

Supplementary Table 2, 3, and 4 list the AUROC, sensitivity, specificity, F1-score, and binary classification accuracy for 14 labels (no finding, enlarged cardiomediastinum, cardiomegaly, airspace opacity, lung lesion, edema, consolidation, pneumonia, atelectasis, pneumothorax, pleural effusion, pleural other, fracture, support devices) in three experiments (internal validation, external validation, end-user scenario). Four MIMIC-based models (*Model-ORIG*, *Model-TRNS*, *Model-PHOT*, and *Model-RECA*) which were tested on two CXR datasets (MIMIC-CXR and CheXpert CXR) and three photograph datasets (*Photo-MMC*, *Photo-CXP*, and *Photo-MED*).

Supplementary Figure 3 shows the results for the three experiments: internal and external validation for four CheXpert-based models (*Model-ORIG*, *Model-TRNS*, *Model-PHOT*, and *Model-RECA*). The AUROCs of different approaches for six thoracic pathologies including cardiomegaly, edema, consolidation, atelectasis, pneumothorax, and pleural effusion are selected due to clinical importance. In Supplementary Figure 3a, the blue line (the model trained and tested on the original CheXpert CXRs) shows the highest AUROC score for each label. The orange line represents the model trained by original CXRs and tested on smartphone photographs, showing a decrease in the performance of the model on the original CXRs. The green line indicates the improvement of the recalibrated model. In Supplementary Figure 3b, we used the smartphone photographs taken from the MIMIC-CXR dataset (*Photo-MIMIC*) as testing data, and the model was trained based on CheXpert datasets. The red line presents the results when the model was trained on *Photo-CXP*. The pink line presents the results when the model was transferred from *Model-ORIG* and fine-tuned on *Photo-CXP*. The models tested on non-photography CXRs (blue lines) are taken as the comparison reference. In summary, the *Model-RECA* has the best performance except for the baseline model, which is consistent with our conclusion in our main research content. Supplementary Table 5 and 6 show the AUROC, sensitivity, specificity, F1-score, and binary classification accuracy of CheXpert-based models for all 14 labels.

In Supplementary Table 7, we compare the model performance on *Photo-MED* when using different hyper-parameter settings determined by random selection, manual selection, and automatic selection. The automatic selection was implemented by maximizing the similarity between the augmented and real CXR photographs. The similarities were computed by complex wavelet structural

similarity method and the Bhattacharyya distance of image histogram. The best performance can be achieved when using automatic selection. However, the similar results using random or manual selection suggested that our protocol is not sensitive to hyper-parameters. Moreover, using similarity comparison not only has the best performance across nearly every label but also saves time for not subjectively guessing values for hyperparameters in use.
